# Neuron‐Derived Vesicular circMagi1 Restrains Vascular Repair After Ischemic Stroke by Promoting TRIM65‐Mediated ANXA2 Degradation

**DOI:** 10.1002/advs.77808

**Published:** 2026-10-15

**Authors:** Youkun Bi, Qun Liu, Baohua Zou, Wenyuan Wang, Xinlong Qiao, Lin Gao, Chaoxu Miao, Ningning Huang, Shuqing Tang, Rui Guo, Changqing Zeng, Ce Jia, Yuanchao Xue, Xuejun Guo, Jianjun Chen, Huiwen Wang, Guangju Ji

**Affiliations:** ^1^ Institute of Biomedical Research Henan Academy of Sciences Zhengzhou China; ^2^ State Key Laboratory of Epigenetic Regulation and Intervention, Institute of Biophysics Chinese Academy of Sciences Beijing China; ^3^ Faculty of Synthetic Biology Shenzhen University of Advanced Technology Shenzhen China; ^4^ School of Pharmacy Yantai University Yantai China; ^5^ College of Life Science Liaoning Normal University Dalian China; ^6^ Yuan Sheng Biotechnology Ltd. Qingdao China; ^7^ Puyang Oilfield General Hospital Affiliated to Henan Medical University Puyang China; ^8^ Department of Cardiac Surgery Peking University Third Hospital Beijing China; ^9^ College of Life Sciences Henan Normal University Xinxiang China

**Keywords:** acute ischemic stroke, angiogenesis, ANXA2, circMagi1, TRIM65

## Abstract

Circular RNAs (circRNAs) contribute to stroke pathogenesis, yet their intercellular functions within the ischemic neurovascular unit remain poorly defined. Profiling of plasma extracellular vesicles (EVs) revealed a higher abundance of circMagi1 in patients with acute ischemic stroke compared with healthy controls. This upregulation was recapitulated in the ischemic mouse brain, where cell‐type‐resolved analyses identified injured neurons as a primary source of circMagi1‐containing EVs that accumulate in endothelial cells. Mechanistically, endothelial circMagi1 binds ANXA2 and enhances its engagement with TRIM65, thereby promoting TRIM65‐mediated ANXA2 ubiquitination and proteasomal degradation and restraining angiogenesis. To therapeutically target this axis in vivo, treatment with siCircMagi1‐RVG‐EVs was initiated on post‐stroke day 1 and maintained thereafter, while siTrim65‐Ang2‐EVs were introduced from either day 1 or day 9. Among the schedules tested, adding siTrim65‐Ang2‐EVs from day 9 was associated with greater neurovascular recovery than sustained circMagi1‐targeted treatment alone or co‐initiation on day 1. Together, these findings delineate a vesicle‐mediated signaling axis linking neuronal injury to impaired vascular repair and support further evaluation of delayed TRIM65 inhibition during sustained circMagi1 suppression.

## Graphical abstract

After ischemic stroke, injured neurons transfer circMagi1 to endothelial cells through extracellular vesicles. Within endothelial cells, circMagi1 binds ANXA2 and enhances its engagement with TRIM65, promoting ANXA2 ubiquitination and proteasomal degradation to constrain angiogenic repair. Later TRIM65 inhibition during sustained circMagi1 suppression is associated with ANXA2 preservation, vascular regeneration, inflammatory resolution, and neurological recovery.

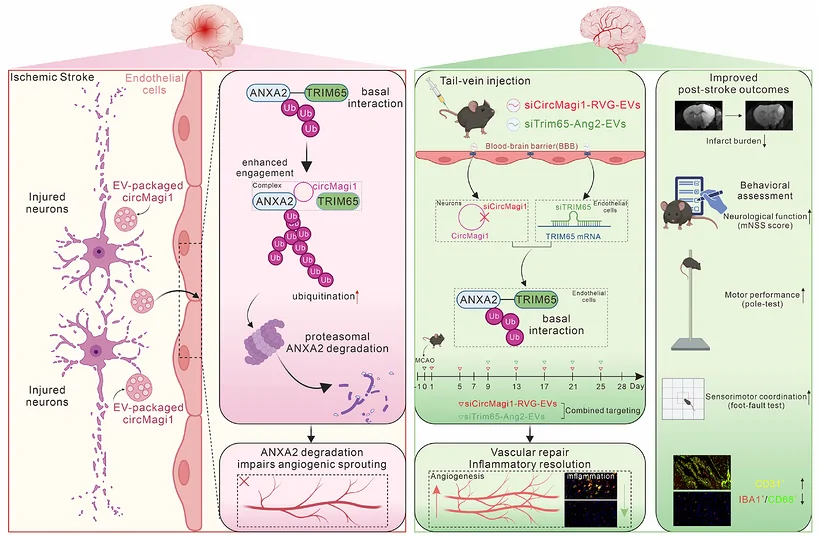

## 1 Introduction

Ischemic stroke remains a leading cause of long‐term disability worldwide and continues to impose a heavy burden on public health systems [1, 2]. Although current standards of care focus on recanalizing large occluded arteries, successful macrovascular restoration often fails to guarantee tissue perfusion due to the “no‐reflow” phenomenon [3]. This persistent microcirculatory failure suggests that functional restitution is not merely a hydraulic problem but a biological one constrained by the limited regenerative capacity of the neurovascular unit (NVU) [4]. Under physiological conditions, neurons and endothelial cells function as an integrated syncytium to match metabolic demand with perfusion [5]. However, ischemic stress profoundly disrupts this homeostatic coupling, converting adaptive signaling into maladaptive crosstalk that actively suppresses tissue repair. Unraveling the precise molecular messengers that mediate this pathological communication is essential for understanding neurovascular dysfunction [6, 7].

Circular RNAs (circRNAs) have emerged as pivotal determinants of stroke pathogenesis [8]. Distinguished by a unique covalently closed structure that confers exceptional stability, these transcripts are uniquely suited to mediate sustained signaling within the neurovascular niche [9]. Recent evidence indicates that extracellular vesicles (EVs) selectively package these stable messengers and shuttle them between compromised cells to dictate survival and inflammatory responses [10]. While the canonical function of circRNAs as microRNA sponges is well documented, emerging structural biology studies reveal a paradigm shift wherein specific circRNAs fundamentally alter cell fate by acting as functional macromolecular scaffolds to rewire protein degradation networks [11, 12, 13]. Yet, the identity of specific pathogenic circRNAs that hijack this endothelial proteolytic machinery to arrest angiogenesis remains a critical knowledge gap [12], obscuring druggable targets that could unlock the latent regenerative capacity of the stroke brain.

Intercepting specific pathogenic circRNAs within the central nervous system presents a formidable translational challenge defined by both spatial barriers and temporal complexity [14]. Although the unique back‐splicing junction (BSJ) of these molecules allows for the design of highly specific nucleic acid therapeutics [11], their in vivo application is severely impeded by the blood‐brain barrier (BBB) [15]. This physiological gatekeeper restricts central access and prevents the precise manipulation of specific cell types within the neurovascular niche [16]. Furthermore, the dynamic evolution of ischemic pathology requires interventions that can dissect signaling events not only at their exact cellular source but also at precise temporal stages. Addressing this multifaceted problem requires advanced nanomedicine strategies that can navigate the BBB to decouple RNA‐mediated crosstalk in distinct cellular compartments [17]. Such precision targeting is critical to bridging the gap between identifying an in vitro molecular interaction and translating it into a timing‐informed therapeutic approach for stroke recovery.

In this study, we identify a vesicle‐mediated circRNA pathway linking neuronal injury to impaired endothelial repair after ischemic stroke. Plasma EV profiling and cell‐resolved analyses in mice implicate injured neurons as a major source of circMagi1‐containing EVs that accumulate in endothelial cells. In endothelial cells, circMagi1 binds ANXA2 and enhances its engagement with TRIM65, thereby promoting ANXA2 ubiquitination and proteasomal degradation and restraining angiogenesis. We further compare the early vs. delayed addition of TRIM65 inhibition during circMagi1 suppression maintained from the early post‐ischemic period, providing a rationale for investigating delayed TRIM65 intervention in neurovascular repair.

## 2 Results

### 2.1 CircMagi1 is Elevated in Circulating EVs During Acute Ischemic Stroke and Induced in Ischemic Brain Tissue

Plasma EVs were isolated and purified from acute ischemic stroke (AIS) patients and healthy controls (HCs), followed by circRNA‐focused RNA sequencing of rRNA‐depleted, RNase R‐treated EV‐packaged RNAs to profile circRNAs based on the BSJ (Figure S1a). Using multiple‐algorithm cross‐validation (STAR, CIRCexplorer2, CIRI2, and DCC) with thresholds of *p <* 0.05 and |log_2_FC| > 1, ten differentially expressed circRNAs were identified between AIS patients and HCs (Figure 1a and Table S1). These candidate circRNAs were validated by qPCR, revealing notable upregulation of circFkbp8, circMagi1, and circNucb1 in AIS patients compared to HCs (*p <* 0.05) (Figure S1b). Expression of the three circRNAs was further examined in plasma EVs from mouse middle cerebral artery occlusion (MCAO) models, with circMagi1 and circNucb1 showing marked upregulation relative to mouse healthy controls (mHCs) (*p <* 0.05) (Figure S1c). CircMagi1 was identified as the most consistently dysregulated transcript in both humans and mice, with 94% sequence homology (Figure 1b and Figure S1d).

**FIGURE 1:**
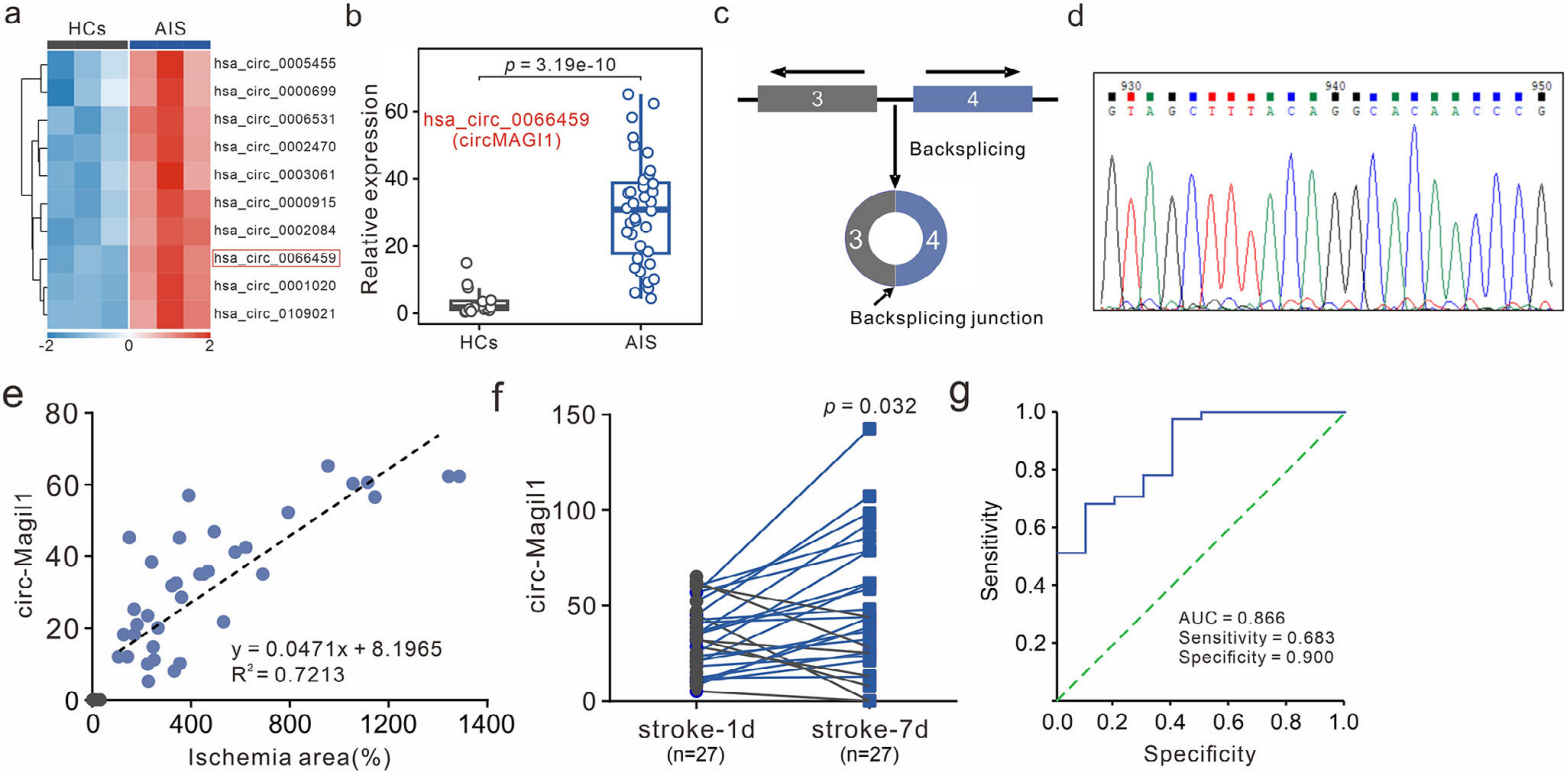
circMagi1 is elevated in plasma EVs from patients with acute ischemic stroke. (a) Hierarchical clustering of the top 10 differentially expressed circRNAs in plasma EVs from patients with AIS and HCs in the discovery cohort. circMagi1 (hsa_circ_0066459) is highlighted. (b) RT‐qPCR validation of plasma EV circMagi1 abundance in patients with AIS (n = 36) and HCs (n = 15). (c) Schematic representation of circMagi1 biogenesis through back‐splicing of exons 3 and 4 of the *MAGI1* gene. (d) Sanger sequencing of the circMagi1 back‐splice junction. (e) Association between plasma EV circMagi1 abundance and MRI‐defined ischemic lesion burden in patients with AIS (n = 36), evaluated by Pearson correlation and simple linear regression. (f) Paired analysis of plasma EV circMagi1 abundance on days 1 and 7 after stroke (n = 27). (g) Receiver operating characteristic curve evaluating the ability of plasma EV circMagi1 to distinguish patients with AIS from HCs. Box plots show the median, interquartile range, and minimum and maximum values. Statistical significance was determined using an unpaired two‐tailed Student's t‐test in (b) and a paired two‐tailed Student's t‐test in (f).

CircMagi1 is circularized through back‐splicing between exons 3 and 4 of the *Magi1* gene, as confirmed by sequencing data at the BSJ (Figure 1c,d). In MCAO mice, TTC (2,3,5‐triphenyltetrazolium chloride) staining quantified post‐stroke infarct area, which correlated strongly with plasma EV‐packaged circMagi1 levels (R^2^ = 0.8347) (Figure S1e,f). Receiver operating characteristic (ROC) analysis demonstrated robust discriminatory performance (AUC = 0.944; Sensitivity = 0.833; Specificity = 1.000) (Figure S1g). In AIS patients, the imaging‐defined ischemic lesion extent showed a strong linear association with plasma EV‐packaged circMagi1 levels (R^2^ = 0.7213) (Figure 1e). Longitudinal sampling indicated a significant increase in circMagi1 levels from day 1 to day 7 after stroke onset (n = 27, *p* = 0.032) (Figure 1f). ROC analysis further confirmed robust discrimination between AIS patients and HCs (AUC = 0.866; Sensitivity = 0.683; Specificity = 0.900) (Figure 1g). Overall, these data identify circMagi1 as a conserved, stroke‐responsive EV‐packaged circRNA that correlates with ischemic lesion burden in both mice and patients and provides robust diagnostic discrimination for AIS.

### 2.2 AAV‐Mediated circMagi1 Silencing Reduces Ischemic Injury and Improves Neurological Recovery

CircMagi1 was broadly upregulated in the ischemic brain compared with sham controls, with the strongest enrichment observed in the peri‐lesional region, reaching approximately 20‐fold higher levels than the corresponding sham brain regions (Figure S2a,b). To assess the functional relevance of this induction, we silenced circMagi1 in the MCAO model using an AAV‐shRNA strategy. AAV‐shCircMagi1 or scramble control vectors were administered by intracerebroventricular injection three weeks before MCAO to ensure sufficient shRNA expression at the time of ischemic injury(Figure 2a and Figure S2c). Successful MCAO induction was defined by a reduction of at least 80% in cerebral blood flow relative to baseline, as measured by laser speckle contrast imaging (Figure S2d). At 24 h after MCAO (day 23), acute lesion burden was evaluated in an endpoint cohort using T2‐weighted magnetic resonance imaging (MRI) and TTC staining. Both representative MRI images and TTC‐stained sections showed smaller ischemic lesions in AAV‐shCircMagi1‐treated mice than in scramble controls (Figure 2b,c).

**FIGURE 2:**
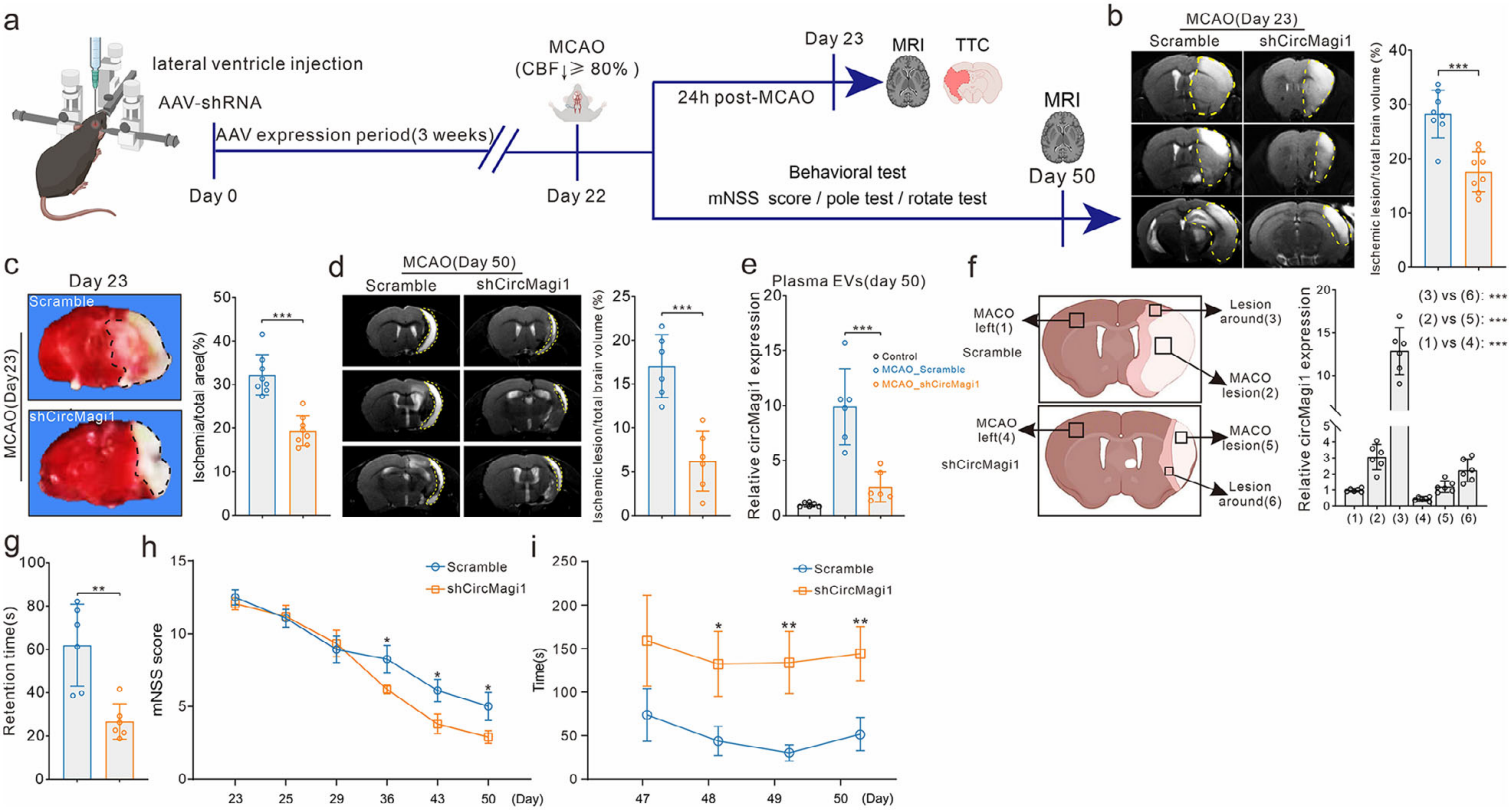
AAV‐mediated circMagi1 silencing reduces ischemic injury and improves neurological recovery. (a) Experimental design. AAV‐shCircMagi1 or scramble control vectors were administered by intracerebroventricular injection 21 days before MCAO. An acute cohort underwent MRI at 24 h after MCAO and was subsequently used for TTC analysis. An independent longitudinal cohort underwent behavioral testing through experimental day 50, followed by endpoint MRI and tissue collection. (b) Representative T2‐weighted MRI images obtained 24 h after MCAO. Lesion volumes were calculated from serial anatomically matched coronal images and normalized to total brain volume (n = 7). (c) Representative TTC‐stained coronal sections from the acute cohort. Quantification shows edema‐corrected infarct volume normalized to contralateral hemisphere volume (n = 7). (d) Representative endpoint T2‐weighted MRI images and quantification of residual lesion burden on day 50 (n = 6). (e) RT‐qPCR analysis of circMagi1 abundance in plasma EVs on day 50 (n = 6). (f) Definition of the sampled contralateral, ischemic‐core, and peri‐infarct regions and corresponding RT‐qPCR analysis of circMagi1 expression on day 50 (n = 6). (g) Pole‐test retention time (n = 6). (h) Modified neurological severity scores at the indicated time points (n = 6). (i) Rotarod latency to fall during experimental days 47–50 (n = 6). Data are mean ± s.d. Statistical significance was determined using an unpaired two‐tailed Student's t‐test in (b‐d, g), one‐way ANOVA with Tukey's multiple‐comparison test in (e), two‐way ANOVA with Tukey's multiple‐comparison test in (f), and two‐way repeated‐measures ANOVA with Tukey's multiple‐comparison test in (h, i). ^*^ *p <* 0.05, ^**^ *p <* 0.01, and ^***^ *p <* 0.001.

In a separate longitudinal cohort, MRI at the experimental endpoint (day 50) showed that AAV‐shCircMagi1 treatment markedly reduced residual ischemic lesion area compared with scramble controls (Figure 2d). Plasma EV circMagi1 abundance was also reduced in the AAV‐shCircMagi1 group at day 50 (Figure 2e). Region‐resolved RT‐qPCR analysis further confirmed circMagi1 suppression across the sampled brain regions, with the most pronounced reduction in the peri‐lesional zone (Figure 2f). In contrast, the corresponding linear Magi1 transcript was not significantly altered in matched regions, supporting the specificity of the circMagi1‐targeting strategy (Figure S2e,f).

Functionally, AAV‐shCircMagi1‐treated mice showed improved performance in the pole test, as indicated by shorter retention times (Figure 2g). They also exhibited progressive neurological recovery, with mNSS scores separating from scramble controls from day 36 onward (Figure 2h). This delayed separation occurred despite pre‐MCAO AAV‐shCircMagi1 delivery and early reduction of lesion burden at 24 h post‐MCAO, indicating that the mNSS difference emerged during the recovery phase rather than reflecting delayed vector expression. During the final four days of testing (days 47–50), rotarod performance was assessed as an endpoint measure of motor coordination rather than as a longitudinal recovery readout. After habituation training, AAV‐shCircMagi1‐treated mice showed longer latencies to fall than scramble controls (Figure 2i). Together, these data show that circMagi1 silencing reduced acute ischemic lesion burden and produced sustained benefits in lesion resolution and neurological recovery after MCAO, with rotarod performance serving as a complementary endpoint measure.

### 2.3 RVG‐EV‐Mediated circMagi1 Silencing Promotes Microvascular Remodeling and Functional Recovery

To circumvent the delayed onset of AAV‐mediated expression and the delivery constraints imposed by the BBB, we established an RVG‐Lamp2b EV platform for in vivo delivery of siCircMagi1 to achieve therapeutic post‐stroke gene silencing [18]. Briefly, HEK293T cells were transfected with the GNSTM‐RVG‐Lamp2b‐GFP plasmid, and culture supernatants were collected 48 h later and purified by density‐gradient ultracentrifugation to obtain RVG‐Lamp2b‐GFP EVs. After quality validation, EVs were electroporated with siCircMagi1 to generate siCircMagi1‐EVs, with scrambled siRNA‐loaded EVs used as negative controls (NC‐EVs). Absolute RT‐qPCR quantification showed that siCircMagi1‐RVG‐EVs contained (2.03 ± 0.76) × 10^12^ copies of siCircMagi1 per 10^10^ particles (Figure S3a and Table S2). Starting on post‐MCAO day 1, mice received tail‐vein injections of siCircMagi1‐EVs or NC‐EVs (1 × 10^11^ particles in 200 µL PBS), repeated every four days for the duration of the indicated treatment window (Figure 3a).

**FIGURE 3:**
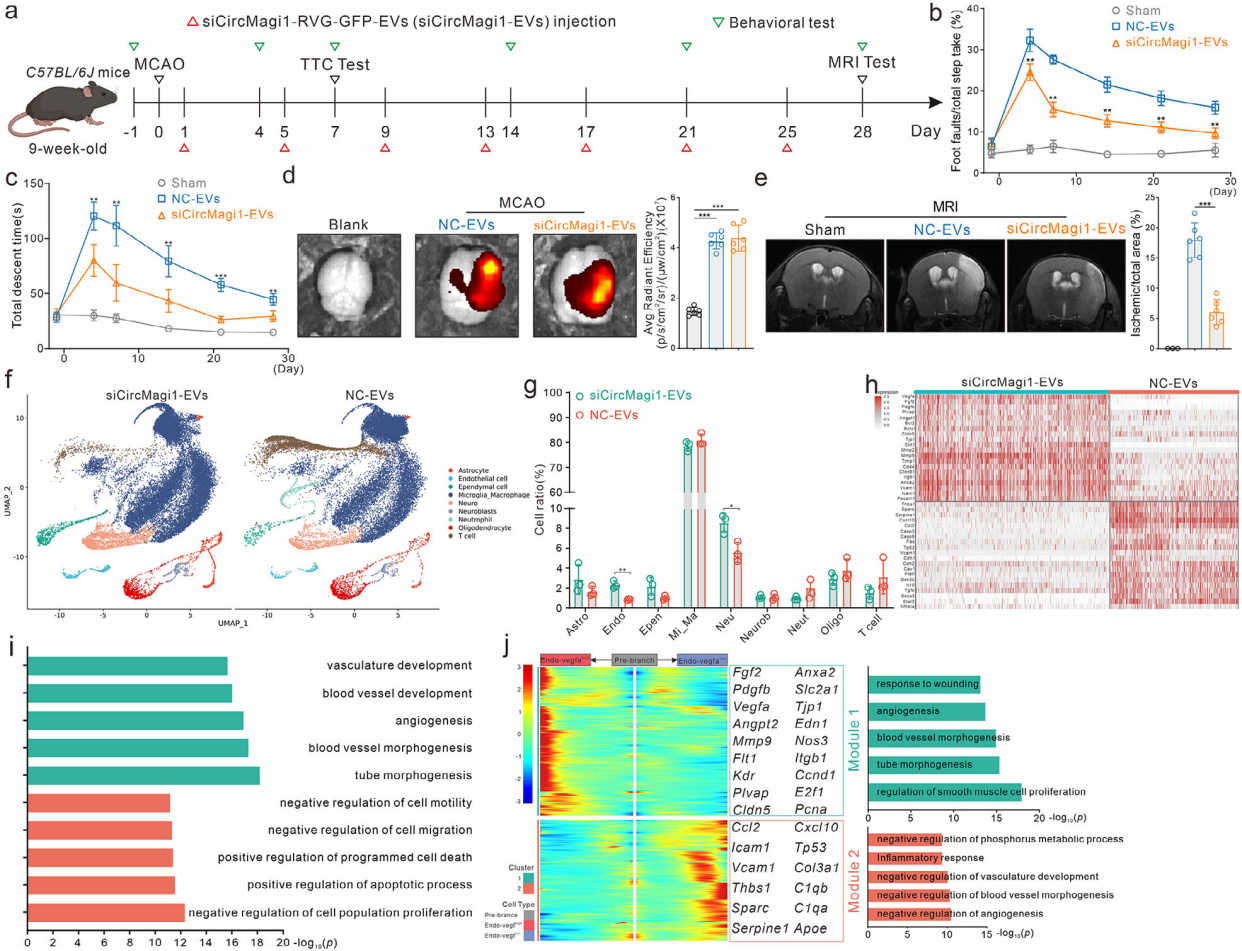
RVG‐EV‐mediated circMagi1 silencing promotes vascular remodeling and functional recovery after MCAO. (a) Experimental timeline for systemic siCircMagi1‐RVG‐EVs administration and longitudinal outcome assessment. (b,c) Foot‐fault performance (b) and pole‐test descent time (c) at the indicated time points (n = 6 mice per group). (d) Ex vivo fluorescence imaging and radiant‐efficiency quantification of GFP‐labeled RVG‐EVs accumulation in the brain (n = 6). (e) Representative T2‐weighted MRI images and quantification of lesion volume on post‐MCAO day 28 (n = 6). (f) UMAP representation of major cell populations identified by single‐cell RNA sequencing of ischemic brain tissue from NC‐EVs‐ and siCircMagi1‐RVG‐EVs‐treated mice (n = 3 biological replicates per group). (g) Relative proportions of the identified cell populations. (h) Heatmap of differentially expressed genes in endothelial cells following siCircMagi1‐RVG‐EVs treatment. (i) Gene Ontology enrichment analysis of endothelial differentially expressed genes. (j) Pseudotime analysis of the endothelial transition from a *Vegfa* ^low^ to a *Vegfa* ^high^ state and the associated gene‐expression modules. Data are mean ± s.d. Statistical significance was determined using two‐way repeated‐measures ANOVA followed by Tukey's multiple‐comparisons test in (b,c), one‐way ANOVA followed by Tukey's multiple‐comparisons test in (d,e), and two‐way ANOVA followed by Šídák's multiple‐comparisons test in (g). ^*^ *p <* 0.05, ^**^ *p <* 0.01, and ^***^ *p <* 0.001.

At day 7 after MCAO, TTC staining revealed a marked reduction in infarct area in siCircMagi1‐EVs‐treated mice relative to NC‐EVs controls, indicating an early attenuation of ischemic tissue injury (Figure S3b). Longitudinal behavioral testing further demonstrated improved post‐ischemic neurological recovery across complementary sensorimotor readouts. Relative to NC‐EVs, siCircMagi1‐EVs reduced contralateral forelimb foot faults in the grid‐walking test (Figure 3b) and improved forelimb‐use symmetry in the cylinder test (Figure S3c). Concordantly, siCircMagi1‐EVs shortened total descent time in the pole test (Figure 3c) and accelerated adhesive removal (Figure S3d), supporting improved sensorimotor integration. At the macroscopic tissue level, ex vivo fluorescence imaging showed comparable signal intensity and distribution between NC‐EVs and siCircMagi1‐EVs, confirming efficient EV entry into the brain (Figure 3d). MRI further corroborated therapeutic benefit and revealed a significant reduction in infarct area in siCircMagi1‐EVs‐treated mice compared with NC‐EVs controls (Figure 3e). Absolute quantification detected siCircMagi1 in the brains of siCircMagi1‐EVs‐treated mice at (5.17 ± 2.07) × 10^9^ copies per mg, and endogenous circMagi1 expression was reduced by ∼80% relative to NC‐EVs controls (Figure S3e,f, and Table S2).

To investigate the cell‐type specific alterations and transcriptional changes underlying the effects of siCircMagi1‐EVs treatment, we performed single‐cell RNA sequencing (scRNA‐seq) on brain tissue from the infarcted hemisphere of mice treated with either NC‐EVs or siCircMagi1‐EVs following MCAO (n = 3 per group). A total of 25 cell clusters were identified using the DoubletFinder and Seurat R packages. Cell types were then assigned based on canonical cell‐type specific markers, revealing nine distinct cell types (Figure 3f). Notably, siCircMagi1‐EVs treatment reconfigured the cellular landscape, with a significant increase of endothelial cells and neurons compared to NC‐EVs (Figure 3g). Given these changes, we focused on endothelial cells due to their pivotal role in vascular remodeling and BBB integrity, both crucial for stroke recovery. Endothelial cell transcriptomic data were extracted, and differentially expressed genes (DEGs) between the two groups were analyzed, with the top DEGs visualized in a heatmap (Figure 3h). GO analysis revealed that the DEGs were primarily enriched in processes related to vascular regeneration and development (Figure 3i).

Vascular endothelial growth factor A (VEGFA) signaling is essential for endothelial cell function, particularly in stroke, where it promotes angiogenesis [19]. In endothelial cells treated with siCircMagi1‐EVs, *Vegfa* expression was notably increased compared to NC‐EVs (Figure S3g). Pseudotime ordering resolved the transcriptional dynamics driving the transition from low to high *Vegfa* expression (*Vegfa^low^*‐to‐*Vegfa^high^*) in endothelial cells. Module 1 encompassed genes upregulated during the *Vegfa^low^*‐to‐*Vegfa^high^* conversion, such as *Fgf2*, *Pdgfa*, *Anxa2*, and *Slc2a1*, primarily contributing to processes such as response to wounding, angiogenesis, and blood vessel morphogenesis. Module 2 contained downregulated genes during the *Vegfa^low^*‐to‐*Vegfa^high^* differentiation, including *Ccl2*, *Cxcl10*, *Icam1*, and *Tp53*, which are involved in regulating inflammatory response, negative regulation of phosphorus metabolic processes, and negative regulation of vasculature development (Figure 3j). The pseudotemporal changes in genes associated with angiogenesis (*Flt1*, *Nos3*), BBB integrity (*Tjp1*), and inflammation (*Icam1*) during the transition from NC‐EVs to siCircMagi1‐EVs highlight the efficacy of siCircMagi1‐EVs treatment in promoting vascular repair (Figure S3h–k).

Collectively, these data show that engineered EVs effectively penetrate the BBB and deliver circMagi1‐silencing siRNA, thereby attenuating cerebral infarction and improving sensorimotor outcomes. Mechanistically, this therapeutic benefit is characterized by the preservation of the endothelial cell pool and the upregulation of a *Vegfa*‐dependent angiogenic transcriptional program.

### 2.4 Injured Neurons Supply circMagi1‐Containing EVs to Endothelial Cells After Ischemic Stroke

To delineate the cellular origin of circMagi1 in the ischemic brain, we isolated neuronal and endothelial fractions from whole brain tissue after MCAO using sequential immunomagnetic sorting (Figure 4a). Flow cytometric validation confirmed the high purity of the isolated fractions, with mean purities of 86.3% ± 2.1% for NeuN^+^ neurons and 91.5% ± 3.2% for CD31^+^ endothelial cells. Representative plots showed 86.7% NeuN^+^ cells and 92.2% CD31^+^ cells (Figure S4a). Longitudinal RT‐qPCR profiling revealed a clear temporal divergence between these populations. circMagi1 increased rapidly in the neuronal fraction as early as 6 h after MCAO, whereas endothelial circMagi1 upregulation was delayed and became evident at later time points (Figure 4b). This temporal sequence indicated that endothelial circMagi1 accumulation may occur secondary to neuronal circMagi1 induction.

**FIGURE 4:**
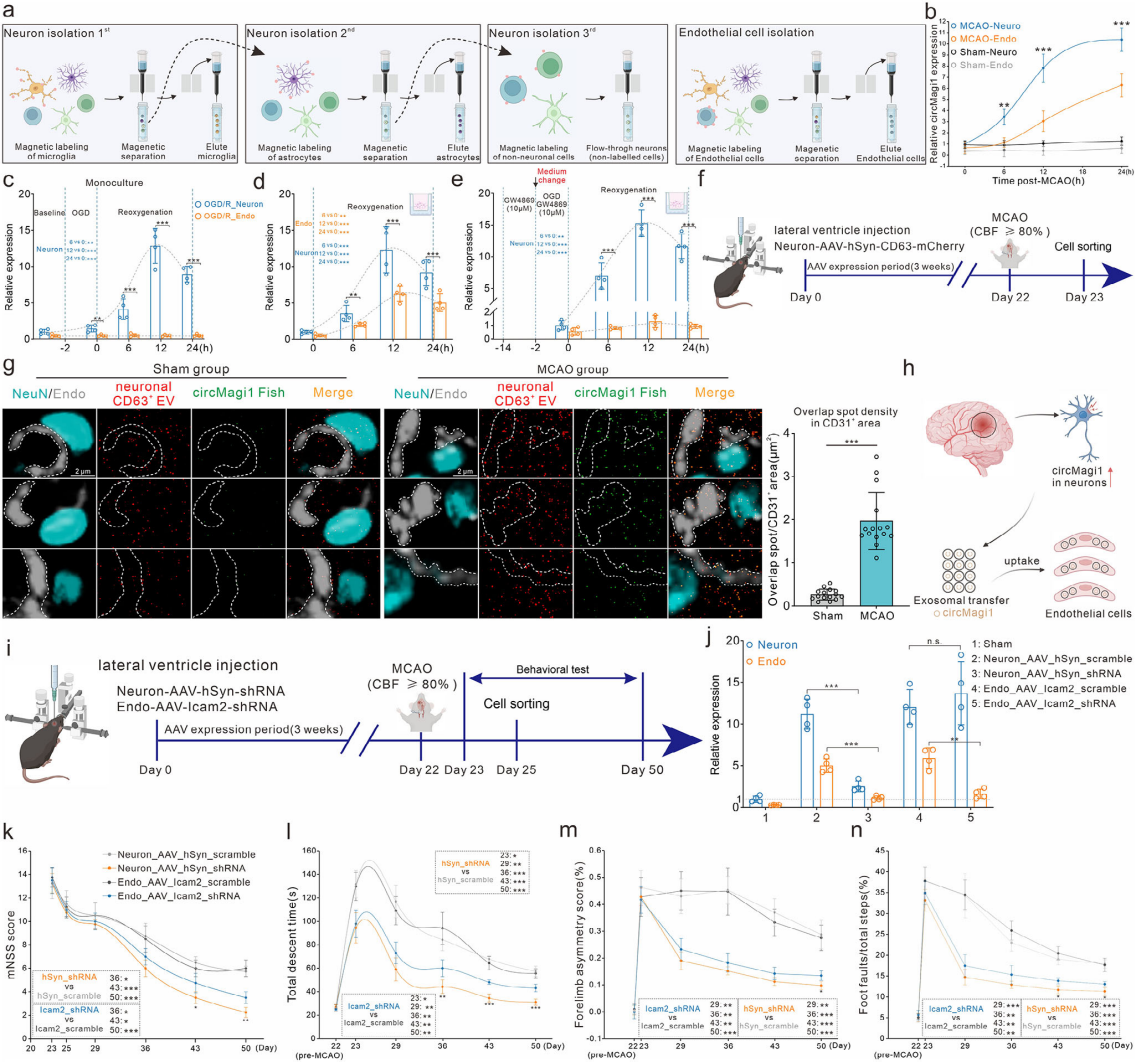
Injured neurons supply circMagi1‐containing vesicular signals to endothelial cells after ischemic stroke. (a) Sequential immunomagnetic isolation of neuronal and endothelial fractions from mouse brain tissue. (b) Time‐course RT‐qPCR analysis of circMagi1 in sorted neurons and endothelial cells after MCAO (n = 4). (c) circMagi1 expression in primary neurons and brain microvascular endothelial cells subjected separately to OGD/R (n = 4 independent cultures). (d) circMagi1 expression in neurons and endothelial cells in a non‐contact transwell co‐culture exposed to OGD/R (n = 4). (e) Effect of the EV‐secretion inhibitor GW4869 on circMagi1 expression in the co‐culture system (n = 4). (f) In vivo neuronal EV‐reporter design. AAV‐hSyn‐CD63‐mCherry was administered intracerebroventricularly 3 weeks before MCAO, and peri‐infarct tissue was collected 24 h after MCAO. (g) Representative fluorescence images showing NeuN‐positive neurons (cyan), CD31‐positive endothelial structures (Endo; gray‐white), neuronal CD63‐mCherry reporter signals (red), circMagi1 FISH puncta (green), and merged signals in sham and MCAO brain sections. White dashed contours delineate CD31‐positive endothelial regions. Quantification shows the density of spatially overlapping CD63‐mCherry and circMagi1 puncta within CD31‐positive areas. Five prespecified imaging fields were analyzed per mouse from three mice in each group. Each point represents one imaging field, yielding 15 field‐level measurements per group. Statistical analysis accounted for the nesting of imaging fields within individual mice. Scale bars, 2 µm. (h) Working model of neuron‐to‐endothelial transfer of circMagi1‐containing vesicular signals. (i) Experimental design for cell‐type‐biased circMagi1 silencing using hSyn‐ or Icam2‐driven AAV vectors. (j) RT‐qPCR verification of circMagi1 knockdown in sorted neuronal and endothelial fractions (n = 4). (k‐n) mNSS (k), pole‐test descent time (l), forelimb‐use asymmetry (m), and foot‐fault rate (n) at the indicated time points (n = 6). Data are mean ± s.d. Statistical significance was determined using two‐way ANOVA with Tukey's multiple‐comparison test in (b‐e, j), an unpaired two‐tailed Student's t‐test in (g), and two‐way repeated‐measures ANOVA with Tukey's multiple‐comparison test in (k‐n). ^*^ *p <* 0.05, ^**^ *p <* 0.01, and ^***^ *p <* 0.001.

To assess cell‐autonomous induction of circMagi1 by ischemic stress, primary neurons and endothelial cells were maintained in separate monocultures and subjected to oxygen‐glucose deprivation followed by reoxygenation (OGD/R). CircMagi1 was robustly induced in neurons during reoxygenation, whereas endothelial cells showed little autonomous induction over the same time course (Figure 4c). In neuron‐endothelial co‐cultures, however, circMagi1 accumulated in endothelial cells after reoxygenation, following the rise observed in neurons (Figure 4d). Inhibition of neutral sphingomyelinase‐dependent EV release with GW4869 markedly reduced endothelial circMagi1 accumulation without preventing neuronal circMagi1 induction (Figure 4e). These data supported neurons as the major ischemia‐responsive source of circMagi1 and implicated EV‐mediated communication in endothelial circMagi1 accumulation.

To further examine this process in vivo, we established a neuronal EV reporter system by intracerebroventricular injection of AAV‐hSyn‐CD63‐mCherry three weeks before MCAO induction (Figure 4f). This design enabled neuron‐derived CD63‐mCherry‐positive EV‐associated signals to be traced after ischemic injury without directly tagging circMagi1. At 24 h after MCAO, peri‐infarct brain sections were subjected to fluorescence in situ hybridization (FISH) for circMagi1 combined with immunofluorescence staining for neuronal and endothelial markers. Compared with sham mice, MCAO markedly increased the density of overlapping neuronal CD63‐mCherry reporter and circMagi1 FISH puncta within CD31^+^ endothelial regions (Figure 4g). Together, the transfer experiments and GW4869‐mediated blockade support a model in which ischemic neurons release circMagi1 in EVs, leading to its accumulation in endothelial cells (Figure 4h).

Because two‐dimensional colocalization alone cannot distinguish surface‐associated signals from internalized material, we next quantified neuronal CD63‐mCherry reporter fluorescence in flow‐sorted CD31^+^ endothelial cells before and after ex vivo acid washing. MCAO increased CD63‐mCherry fluorescence in the endothelial fraction. Acid washing partially reduced this signal, indicating a surface‐associated component, whereas the residual fluorescence remained significantly higher than that in sham controls (Figure S4b). Similarly, RT‐qPCR using divergent primers spanning the circMagi1 back‐splice junction and normalization to an exogenous circular RNA spike‐in detected increased circMagi1 abundance in sorted CD31^+^ endothelial cells after MCAO. This enrichment was attenuated, but not abolished, by acid washing (Figure S4c). Together with the in situ colocalization data, these analyses support a model of neuron‐to‐endothelial vesicular transfer of circMagi1 after ischemic injury, with the acid‐wash‐resistant signals consistent with intracellular accumulation.

To examine the functional contribution of neuron‐derived circMagi1, we delivered hSyn‐ or Icam2‐driven AAV‐shRNA [20, 21] by intracerebroventricular injection to preferentially silence circMagi1 in neurons or endothelial cells, respectively (Figure 4i). hSyn‐driven silencing reduced circMagi1 abundance in both neuronal and endothelial fractions, whereas Icam2‐driven silencing predominantly reduced endothelial circMagi1 with minimal effect on the neuronal fraction (Figure 4j). Both interventions improved neurological recovery relative to their respective scramble controls; however, hSyn‐driven silencing produced greater and more sustained reductions in mNSS scores, pole‐test descent time, forelimb‐use asymmetry, and foot‐fault rate (Figure 4k–n). Collectively, these findings identify injured neurons as a major source of circMagi1 that accumulates in endothelial cells through EV‐mediated transfer and show that neuron‐biased circMagi1 silencing promotes greater long‐term neurological recovery than endothelial‐biased silencing.

### 2.5 CircMagi1 Binds ANXA2 and Suppresses Endothelial Angiogenic Function

While our in vivo findings substantiate the therapeutic potential of circMagi1 silencing, the molecular effectors executing its endothelial pathogenicity remain undefined. To systematically deconvolute this mechanism, we characterized the circMagi1 interactome via RNA antisense purification (RAP) using probes specific to its unique backsplicing junction (Figure S5a). Silver staining confirmed the specificity of the pull‐down (Figure S5b), and subsequent liquid chromatography‐tandem mass spectrometry (LC‐MS/MS) analysis revealed a predominant enrichment of proteins governing angiogenesis, tight junction integrity, and inflammatory responses (Figure S5c and Table S3). To pinpoint the bona fide functional mediator, we cross‐referenced these physical interactors with the top 20 driver genes defining the endothelial pseudotime trajectory. This multi‐omics convergence strategy highlighted ANXA2 as the leading candidate (Figure 5a).

**FIGURE 5:**
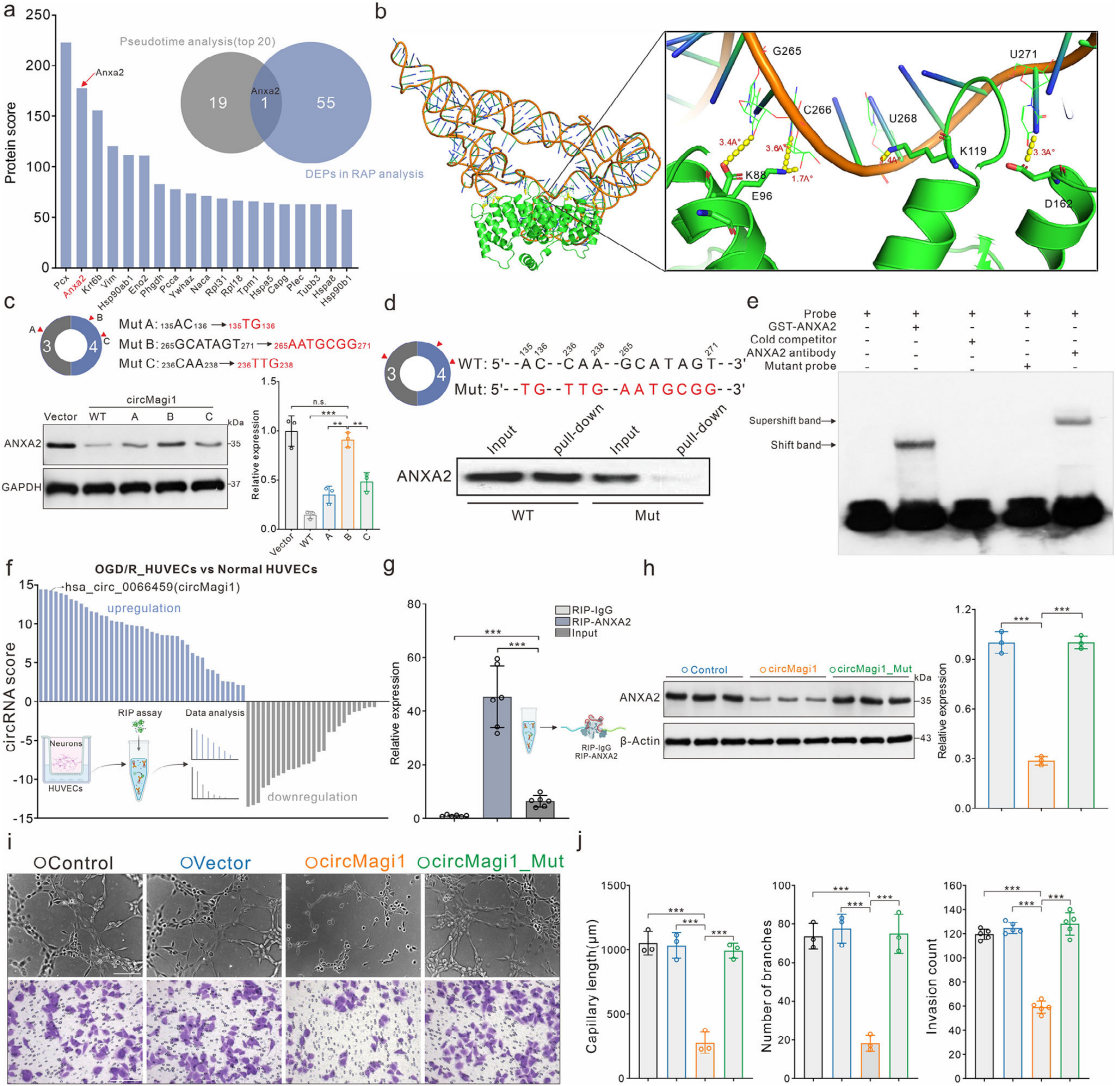
circMagi1 binds ANXA2 and suppresses endothelial angiogenic function. (a) Identification of ANXA2 as a candidate circMagi1‐associated protein by RNA antisense purification coupled with mass spectrometry (RAP‐MS). The bar graph shows proteins identified by RAP‐MS, and the inset shows the overlap between their corresponding genes and genes associated with the endothelial pseudotime trajectory. (b) Structural model of the circMagi1‐ANXA2 complex, highlighting predicted contacts involving the circMagi1 G265‐U271 region. (c) ANXA2 protein abundance in HUVECs expressing WT circMagi1 or variants carrying mutations in predicted ANXA2‐interacting regions. (d) RNA antisense purification followed by ANXA2 immunoblotting using WT circMagi1 or the combined interface mutant circMagi1_Mut. (e) EMSA of recombinant GST‐ANXA2 and biotinylated circMagi1 probes, including cold‐competitor, mutant‐probe, and antibody‐supershift controls. (f) RIP‐seq ranking of circRNAs recovered in ANXA2 immunoprecipitates from OGD/R‐treated HUVECs. (g) RT‐qPCR validation of circMagi1 enrichment in ANXA2 immunoprecipitates relative to matched IgG controls (n = 3). (h) ANXA2 abundance in HUVECs expressing control vector, WT circMagi1, or circMagi1_Mut. β‐actin served as the loading control. (i) Representative tube‐formation and transwell‐invasion images. Scale bars, 100 µm. (j) Quantification of total tube length, branch number, and invaded cells (n = 3 independent experiments). Data are mean ± s.d. Statistical significance was determined using a paired two‐tailed Student's t‐test in (g) and one‐way ANOVA with Tukey's multiple‐comparison test in (c, h, j). ^*^ *p <* 0.05, ^**^ *p <* 0.01 and ^***^ *p <* 0.001.

To elucidate the structural basis of this interaction, we employed AlphaFold algorithms to predict the molecular docking interface, revealing three potential binding motifs within the circMagi1 sequence. The primary interface features a robust network of hydrogen bonds and electrostatic interactions anchoring ANXA2 residues K88, E96, K119, and D162 to a specific nucleotide motif at positions 265–271 (G265‐U271) (Figure 5b). Two subsidiary binding pockets were also identified (Figure S5d), characterized by interactions between the ANXA2 G207/T208/K249/D166 cluster and nucleotides A135‐C136, alongside the V51/D326/K329 motif docking with the C236‐A238 region. To empirically validate these predictions, we generated site‐specific variants (Mut A, Mut B, and Mut C). Western blot analysis confirmed that the 265–271 motif (Mut B) is required for the observed effect. While wild‐type circMagi1 markedly reduced ANXA2 protein abundance, this suppression was largely abolished in the Mut B variant, which restored Anxa2 expression to baseline levels (Figure 5c, original blot is provided in Figure S8a). In contrast, mutations at alternative sites (Mut A and Mut C) largely preserved inhibitory capacity, establishing the Mut B region as the dominant structural determinant governing this interaction. Finally, to confirm direct physical engagement, we performed an RNA pull‐down assay using a combined mutant disrupting all three predicted interfaces. Immunoblotting revealed that while wild‐type circMagi1 robustly enriched Anxa2, this interaction was completely abrogated in the mutant (Figure 5d, original blot is provided in Figure S8b), supporting the sequence specificity of the circMagi1‐ANXA2 interaction.

Next, we substantiated direct physical interaction using electrophoretic mobility shift assays (EMSA) with recombinant GST‐ANXA2. The biotinylated circMagi1 probe yielded a specific mobility shift that was displaceable by unlabeled probe but not by the mutant, while an anti‐ANXA2 antibody induced a supershift (Figure 5e, original blot is provided in Figure S8c). Reciprocally, RNA immunoprecipitation (RIP) assays in OGD/R‐treated endothelial cells identified circMagi1 (hsa_circ_0066459) as a prominently enriched ANXA2 partner (Figure 5f and Table S4), with quantitative confirmation provided by RT‐qPCR (Figure 5g). To link this physical engagement to regulatory function, we examined ANXA2 protein abundance. Western blotting showed that WT circMagi1 markedly reduced ANXA2 abundance, whereas circMagi1_Mut failed to reproduce this effect (Figure 5h, original blot is provided in Figure S8d). Given ANXA2's established pro‐angiogenic role [22], we next examined endothelial behavior. Wild‐type circMagi1 significantly compromised angiogenesis and invasion, evidenced by marked reductions in capillary length, number of branches, and invasion count. Crucially, these inhibitory phenotypes were effectively nullified in the mutant group (Figure 5i,j).

In conclusion, our structural and biochemical characterization defines circMagi1 as a direct ligand for ANXA2, driven by specific molecular docking determinants. We demonstrate that this physical engagement is the mechanistic prerequisite for biological activity, as disrupting the binding interface prevents circMagi1‐mediated ANXA2 depletion and the subsequent impairment of endothelial function.

### 2.6 circMagi1 Promotes TRIM65‐Mediated ANXA2 Ubiquitination by Enhancing ANXA2‐TRIM65 Engagement

CircRNAs are known to modulate target protein stability through post‐translational mechanisms, particularly the ubiquitin‐proteasome system (UPS) and autophagy pathways [8, 14]. To determine whether circMagi1 regulates ANXA2 protein stability, we monitored ANXA2 turnover using cycloheximide (CHX) chase assays [23]. CircMagi1 overexpression markedly accelerated ANXA2 decay compared with vector controls. This accelerated loss was rescued by the proteasome inhibitor MG132 [24], whereas chloroquine (CQ) [25] did not restore ANXA2 abundance (Figure 6a, original blot is provided in Figure S8e). We further examined ANXA2 levels after lysosomal/autophagy blockade with CQ or Bafilomycin A1 (BafA1) [26]. Although these treatments effectively blocked autophagic flux, as indicated by LC3‐II and p62 accumulation, they failed to rescue ANXA2 levels (Figure 6b, original blot is provided in Figure S8f). These results indicate that circMagi1 promotes ANXA2 degradation primarily through the UPS rather than the lysosomal/autophagy pathway.

**FIGURE 6:**
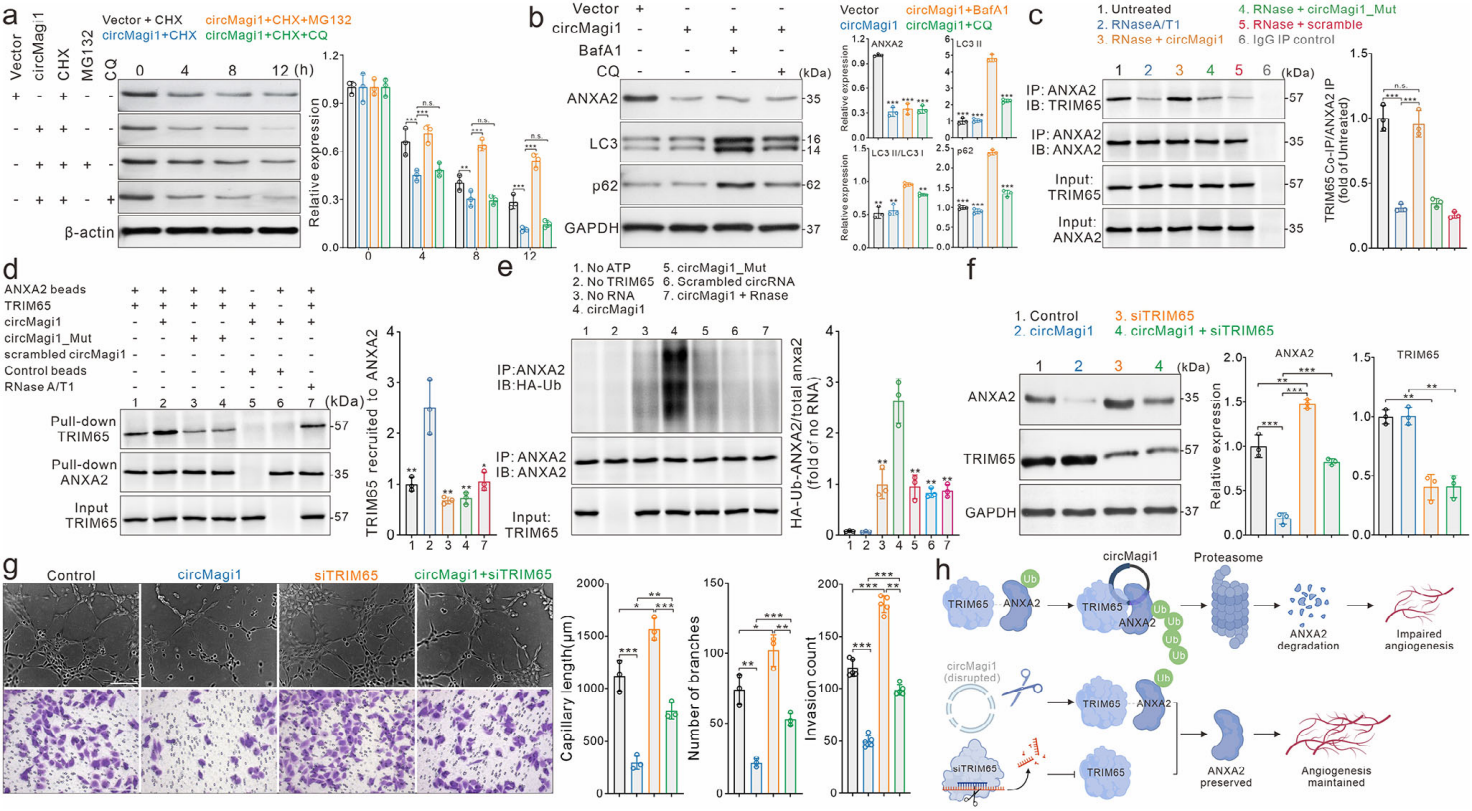
circMagi1 enhances ANXA2‐TRIM65 engagement and promotes TRIM65‐mediated ANXA2 ubiquitination. (a) CHX chase analysis of ANXA2 turnover in vector‐control or circMagi1‐overexpressing HUVECs. All groups were treated with CHX (100 µg/mL) for the indicated durations, with MG132 (10 µM) or CQ (50 µM) added to the indicated circMagi1‐overexpressing groups. (b) ANXA2, LC3, and p62 abundance in circMagi1‐overexpressing HUVECs treated with bafilomycin A1 (BafA1; 10 nM) or CQ (50 µM). (c) RNase‐rescue co‐immunoprecipitation assay examining the contribution of intact RNA to circMagi1‐mediated enhancement of the basal ANXA2‐TRIM65 association. Following RNase A/T1 removal or inactivation, purified WT circMagi1, circMagi1_Mut, or scrambled circRNA was added before ANXA2 immunoprecipitation. (d) Reconstitution of ANXA2‐dependent TRIM65 recruitment using immobilized ANXA2, purified TRIM65, and the indicated RNA constructs. (e) Cell‐free ANXA2 ubiquitination using purified ANXA2, TRIM65, E1, E2, ATP, and HA‐tagged K48‐only ubiquitin. Reactions lacking ATP, TRIM65, or RNA and reactions containing circMagi1_Mut, scrambled circRNA, or RNase are shown. (f) ANXA2 and TRIM65 abundance in HUVECs expressing control vector, circMagi1, siTRIM65, or circMagi1 plus siTRIM65. (g) Representative tube‐formation and transwell‐invasion images and corresponding quantification. Scale bars, 100 µm. (h) Working model in which circMagi1‐bound ANXA2 exhibits increased TRIM65 engagement, leading to ANXA2 ubiquitination, proteasomal degradation, and impaired angiogenic function. Data are mean ± s.d. from three independent experiments. Statistical significance was determined using two‐way ANOVA with Tukey's multiple‐comparison test in (a) and one‐way ANOVA with Tukey's multiple‐comparison test in (b‐g). ^*^ *p <* 0.05, ^**^ *p <* 0.01, and ^***^ *p <* 0.001.

TRIM65 has been reported to function as an E3 ubiquitin ligase for ANXA2 and to promote its proteasomal degradation [27, 28]. We therefore examined whether TRIM65 mediates circMagi1‐induced ANXA2 ubiquitination. In HUVECs expressing HA‐tagged ubiquitin, circMagi1 increased high‐molecular‐weight HA‐Ub‐conjugated ANXA2 after ANXA2 immunoprecipitation. This increase was markedly reduced by TRIM65 knockdown (Figure S6a, original blot is provided in Figure S8g), placing TRIM65 downstream of circMagi1 in ANXA2 ubiquitination. Co‐immunoprecipitation (Co‐IP) analysis further showed that ANXA2 associated with TRIM65 at a basal level, and that increasing circMagi1 expression progressively increased the amount of TRIM65 recovered with ANXA2 (Figure S6b, original blot is provided in Figure S8h). Thus, circMagi1 strengthened the pre‐existing ANXA2‐TRIM65 enzyme‐substrate association.

We next tested whether this enhancement required intact circMagi1. RNase A/T1 treatment reduced TRIM65 recovery in ANXA2 immunoprecipitates. After RNase removal or inactivation, purified WT circMagi1 restored ANXA2‐associated TRIM65, whereas circMagi1_Mut and scrambled circRNA did not (Figure 6c, original blot is provided in Figure S8i). We then reconstituted TRIM65 recruitment using purified components. Immobilized ANXA2 beads captured basal levels of TRIM65, whereas WT circMagi1 markedly increased TRIM65 recruitment to ANXA2. This increase was lost with circMagi1_Mut, scrambled circRNA, or RNase treatment (Figure 6d, original blot is provided in Figure S8j). These findings indicate that intact circMagi1 enhances the basal ANXA2‐TRIM65 interaction through an RNA‐dependent proximity effect.

We next asked whether enhanced TRIM65 recruitment was sufficient to increase ANXA2 ubiquitination in a reconstituted system. Cell‐free ubiquitination reactions were assembled with purified ANXA2, TRIM65, E1, E2, ATP, and HA‐ubiquitin. WT circMagi1 markedly increased HA‐Ub‐conjugated ANXA2, whereas omission of ATP or TRIM65 abolished the ubiquitination signal. In contrast, circMagi1_Mut, scrambled circRNA, and RNase treatment failed to reproduce the WT circMagi1‐induced increase in ANXA2 ubiquitination (Figure 6e, original blot is provided in Figure S8k). These results show that intact circMagi1 is sufficient to enhance TRIM65‐mediated ANXA2 ubiquitination in a purified biochemical system.

Consistent with this mechanism, TRIM65 knockdown restored ANXA2 protein abundance in circMagi1‐overexpressing endothelial cells (Figure 6f, original blot is provided in Figure S8l). Functionally, circMagi1 overexpression impaired endothelial tube formation and invasion, whereas TRIM65 knockdown rescued these defects, restoring capillary length, branch number, and invasion capacity (Figure 6g). Together, these findings support a model in which circMagi1 directly binds ANXA2 and enhances the basal ANXA2‐TRIM65 interaction through an RNA‐dependent proximity effect. This promotes TRIM65‐mediated ANXA2 ubiquitination and proteasomal degradation, thereby restraining endothelial angiogenic function (Figure 6h).

### 2.7 Delayed Addition of TRIM65 Inhibition is Associated With Greater Recovery During Sustained circMagi1 Suppression

TRIM65 contributes to early innate immune regulation through VCAM‐1 degradation and suppression of NLRP3 inflammasome assembly [29], raising the possibility that its inhibition may have different effects depending on when treatment is introduced after ischemic injury. We therefore compared TRIM65 inhibition initiated on post‐MCAO Day 1 or Day 9 during circMagi1 suppression maintained from Day 1 onward. Day 1 and Day 9 were operationally designated as the early acute and subacute initiation points, respectively. siTrim65 was delivered using Angiopep‐2‐modified EVs (Ang2‐EVs) selected for blood‐brain barrier penetration. The siTrim65 mimics contained 2’‐O‐methyl and 2’‐fluoro modifications, phosphorothioate linkages, and a 3’‐cholesterol moiety to enhance nuclease resistance and membrane permeability [30] (Figure 7a). Quantitative analysis showed an encapsulation density of (1.14 ± 0.998) × 10^12^ copies per 10^10^ particles (Figure 7b and Table S5).

**FIGURE 7:**
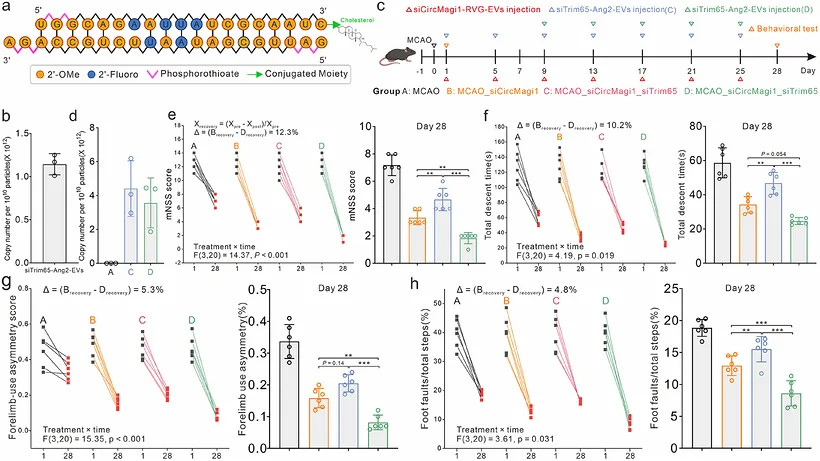
Delayed TRIM65 inhibition during sustained circMagi1 suppression is associated with greater neurovascular recovery. (a) Chemical design of the siTrim65 mimic, showing 2′‐O‐methyl, 2′‐fluoro, phosphorothioate, and 3′‐cholesterol modifications. (b) Absolute quantification of siTrim65 loading in siTrim65‐Ang2‐EVs, expressed as copy number per 10^10^ EV particles (n = 3). (c) Experimental treatment schedules. Group A received MCAO without EV treatment. Group B received siCircMagi1‐RVG‐EVs every 4 days from post‐MCAO day 1 through day 25. Groups C and D received the same siCircMagi1‐RVG‐EV regimen, together with siTrim65‐Ang2‐EVs administered every 4 days from day 1 or day 9 through day 25, respectively. Groups C and D consequently received seven and five siTrim65‐Ang2‐EV administrations, respectively. (d) siTrim65 abundance in ischemic brain tissue from Groups A, C, and D on day 28 (n = 3). (e–h) Individual paired measurements on post‐MCAO Days 1 and 28 and corresponding Day 28 group values for mNSS (e), pole‐test descent time (f), forelimb‐use asymmetry test (g), and foot‐fault test (h) (n = 6 mice per group). Δ represents the descriptive difference in residual behavioral fractions between Groups B and D and was calculated as ∆ = [(X_B, 28_/X_B, 1_)‐(X_D, 28_/X_D, 1_)] × 100. Positive values indicate greater behavioral improvement in Group D. Behavioral changes were analyzed using two‐way repeated‐measures ANOVA, including treatment, time, and the treatment × time interaction. Day 28 group comparisons were analyzed using one‐way ANOVA followed by Tukey's multiple‐comparison test. ^*^ *p <* 0.05, ^**^ *p <* 0.01, and ^***^ *p <* 0.001.

Mice were assigned to four experimental groups (Figure 7c). Group A served as the MCAO control, whereas Group B received siCircMagi1‐RVG‐EVs every four days from Day 1 through Day 25. Groups C and D received the same siCircMagi1‐RVG‐EV regimen, with siTrim65‐Ang2‐EVs administered every four days from Day 1 or Day 9 through Day 25, corresponding to seven and five administrations, respectively. Endpoint analysis detected cerebral siTrim65 under both schedules, reaching (4.41 ± 1.34) × 10^9^ copies in Group C and (3.57 ± 1.20) × 10^9^ copies in Group D (Figure 7d and Table S5). Plasma profiling on Day 28 further showed lower concentrations of IL‐6, TNF‐α, IL‐1β, IFN‐β, C3a, and C5a in Group D than in Group C (Figure S7a), indicating that initiating siTrim65‐Ang2‐EV administration on Day 9 was not associated with an additional elevation of these immune markers at the experimental endpoint.

Behavioral changes between post‐MCAO Days 1 and 28 differed among treatment groups, with significant treatment × time interactions for mNSS scores (F(3, 20) = 14.37, *p <* 0.001), pole‐test descent time (F(3, 20) = 4.19, *p* = 0.019), forelimb‐use asymmetry scores (F(3, 20) = 15.35, *p <* 0.001), and foot‐fault rates (F(3, 20) = 3.61, *p* = 0.031). Descriptive analysis of residual behavioral fractions showed lower Day 28‐to‐Day 1 ratios in Group D than in Group B. The corresponding differences between Groups B and D were 12.3, 10.2, 5.3, and 4.8 percentage points for mNSS, pole‐test descent time, forelimb‐use asymmetry, and foot‐fault rate, respectively (Figure 7e–h). At Day 28, Group D showed significantly lower mNSS scores, forelimb‐use asymmetry scores, and foot‐fault rates than Group B. Pole‐test descent time showed a similar numerical reduction but did not reach statistical significance after Tukey adjustment (adjusted‐*p* = 0.054). Group D also showed significantly lower Day 28 values than Group C across all four outcomes. Conversely, Group C showed higher mNSS scores, pole‐test descent times, and foot‐fault rates than Group B, whereas forelimb‐use asymmetry did not differ significantly between these groups (Table S6). Peri‐infarct analysis further showed that Group D had the highest ANXA2 and CD31 signals, together with reduced TRIM65 expression and lower IBA1 and CD68 signals relative to Group C (Figure S7b,c).

Collectively, Group D showed the most favorable integrated outcome profile among the regimens examined, combining greater ANXA2 preservation and peri‐infarct vascular remodeling with lower neuroinflammatory and endpoint plasma immune‐marker levels and improved functional recovery. These findings support further evaluation of delayed TRIM65 inhibition during sustained circMagi1 suppression.

## 3 Discussion

Restoring microvascular perfusion is a prerequisite for functional recovery after ischemic stroke, yet the molecular signals that restrain vascular repair remain incompletely defined [1, 2, 3]. Here, we identify a pathogenic neuron‐to‐endothelium signaling axis in which injured neurons supply EV‐packaged circMagi1 to endothelial cells, thereby imposing a post‐translational brake on angiogenic recovery. This axis connects neuronal injury with endothelial dysfunction through TRIM65‐mediated ANXA2 degradation, revealing a mechanism by which the ischemic neurovascular unit actively suppresses its own regenerative capacity. Therapeutic experiments further suggest that the effects of downstream TRIM65 inhibition vary across dosing schedules during sustained circMagi1 suppression, highlighting this axis as a candidate target for vascular repair after ischemic stroke.

Our findings suggest a maladaptive inversion of neurovascular coupling. Under physiological conditions, neuron‐endothelial communication supports vascular homeostasis, whereas after ischemic injury, neurons appear to transmit an inhibitory RNA signal that limits endothelial repair [31]. Unlike the canonical miRNA‐sponge function often attributed to circRNAs, circMagi1 acts as an RNA effector that binds ANXA2 and enhances the association between ANXA2 and TRIM65, thereby facilitating ANXA2 ubiquitination and degradation. Structural modeling and mutational analyses identified a circMagi1 region involved in ANXA2 recruitment, suggesting a potential interface for disrupting this pathogenic RNA‐protein interaction [32]. However, because this region is shared with the linear host transcript, direct targeting could risk interfering with physiological *Magi1* expression. We therefore prioritized the unique back‐splice junction as the therapeutic target, enabling selective suppression of the circular RNA while preserving the linear transcript [33].

A remaining challenge is how to define neuron‐to‐endothelium circRNA transfer in the intact brain [34]. Our data support the transfer of neuron‐derived circMagi1 signals to endothelial cells, although they do not track individual endogenous circMagi1 molecules in real time during cell‐to‐cell movement. This is a practical limitation when studying native circRNAs in vivo. We therefore interpret the transfer evidence as a coherent body of support from cell‐type‐specific circMagi1 dynamics, EV‐release blockade, neuronal EV reporter tracing, and surface‐stripping‐resistant endothelial signals, rather than as definitive single‐molecule lineage tracing. Future Cre‐LoxP cargo‐transfer systems [35] or MS2‐tagged circRNA reporters [36] could provide complementary validation, but Cre‐LoxP mainly reports functional cargo delivery, whereas MS2 tagging may alter circRNA packaging or localization. Thus, the most conservative conclusion is that ischemic neurons are a major source of EV‐packaged circMagi1 signals that accumulate in endothelial cells after stroke.

Dissecting this intercellular axis in vivo also required spatially biased manipulation within the central nervous system [37]. RVG‐ and Angiopep‐2‐modified EVs were used not as perfectly cell‐exclusive vehicles, but as delivery platforms designed to enrich intervention at different compartments of the neurovascular axis [18, 38]. Previous studies have shown that RVG modification can enhance CNS delivery of EV or siRNA cargos through acetylcholine receptor‐associated mechanisms, providing a rationale for neuronal or CNS‐biased delivery. Similarly, Angiopep‐2 engages LRP1‐mediated transport across the blood‐brain barrier, and the reported abundance of LRP1 in brain microvascular endothelial cells supports its use for vascular‐compartment‐biased delivery [39]. However, neither targeting mechanism is expected to confer absolute cell‐type specificity in vivo. The dual‐EV design was therefore intended to bias intervention toward the neuronal and vascular compartments of the circMagi1 signaling axis. Within this boundary, the intervention data suggest potential value in coordinating suppression of the upstream neuronal circMagi1 signal with preservation of endothelial ANXA2 during vascular remodeling. Future studies using labeled therapeutic EVs, matched dye and unmodified EV controls, receptor‐competition assays, and quantitative colocalization with NeuN, CD31, and GFAP will be required to define their precise cellular uptake profiles in the ischemic brain.

The dynamic evolution of ischemic pathology implies that downstream pathway inhibition depends on treatment timing and biological context [2, 3, 37]. Here, introducing siTrim65‐Ang2‐EVs on Day 9 during sustained circMagi1 suppression was associated with greater functional and vascular recovery over monotherapy or Day 1 co‐administration, supporting ANXA2 preservation during active vascular remodeling. However, differing treatment durations and cumulative exposures preclude strictly disentangling temporal alignment from drug dosage effects. Furthermore, evaluating TRIM65 timing against a fixed circMagi1 suppression background rather than independent therapeutic phases warrants future dose‐matched studies to define a discrete subacute window. Although Day 28 plasma profiling showed no elevation in inflammatory, interferon, or complement markers, transient immune fluctuations from repeated EV dosing cannot be excluded without serial sampling. Resolving these temporal, exposure‐dependent, and immunological variables will require time‐course and factorial designs.

Beyond its mechanistic implications, the enrichment of EV‐packaged circMagi1 in the plasma of patients with stroke suggests potential value for clinical biomonitoring. Its positive association with infarct volume indicates that circulating EV circMagi1 abundance may reflect ischemic injury burden. The intrinsic resistance of circular RNAs to exonuclease‐mediated degradation, together with their protection within the vesicular lipid bilayer, may favor their stability in circulation [40]. Plasma EV circMagi1 could therefore complement existing approaches to patient stratification and disease monitoring. Its diagnostic and prognostic value will nevertheless require validation in larger independent cohorts, across different sampling times, and in patients with heterogeneous stroke subtypes and comorbidities.

Despite the mechanistic insights provided by this study, translation of these findings remains constrained by interspecies heterogeneity, sex differences, and anatomical complexity. The circMagi1‐TRIM65‐ANXA2 axis was examined primarily in otherwise healthy male mice subjected to MCAO, which does not fully reproduce the vascular fragility associated with aging and comorbidities such as hypertension and diabetes [41]. The absence of female animals is an additional limitation, as sex‐dependent differences in lesion evolution, neurovascular repair, immune activation, and treatment responses may influence both the magnitude and kinetics of benefit. Furthermore, the lissencephalic rodent brain lacks the complex white matter architecture of the human brain, in which microvascular ischemic injury may evolve differently [42]. Although neurons emerged as a major source of circMagi1‐containing EVs, the contributions of pericytes, microglia, and other components of the neurovascular unit remain to be defined [43]. The combined intervention also requires further safety and dose‐schedule evaluation because TRIM65 participates in RIG‐I signaling and innate antiviral immunity [28, 29]. Future studies using sex‐balanced cohorts, dose‐matched treatment schedules, and larger gyrencephalic models will be required to establish the safety, scalability, and generalizability of this therapeutic approach.

## 4 Methods

### 4.1 Human Studies and Animal Experiments

The clinical study protocol was approved by the Ethics Committee of the Puyang Oilfield General Hospital (Approval No. 2021‐03‐0003‐E01) and conducted in strict accordance with the Declaration of Helsinki. Written informed consent was obtained from all participants or their legal proxies prior to enrollment. A total of 36 patients diagnosed with AIS and 15 age‐ and sex‐matched HCs were recruited for this study. AIS diagnosis was established based on comprehensive clinical evaluation and confirmed by MRI. Patients presenting with malignancies, active infections, or concurrent neurological disorders were excluded to minimize confounding factors. Peripheral blood samples were collected in EDTA‐coated tubes from all participants. Subsequently, plasma was separated by centrifugation at 2,000 × g for 10 min at 4°C and immediately stored at −80°C until EVs isolation.

All animal procedures complied with the NIH Guide for the Care and Use of Laboratory Animals and were approved by the Animal Ethics Committee of Henan Normal University (Approval No. HNSD‐2023BS‐0913). Adult male C57BL/6J mice (8 weeks old, 22–25 g) were maintained under specific pathogen‐free conditions. Mice were randomly assigned to experimental groups, and investigators were blinded to group allocation during surgery and outcome assessment. Transient focal cerebral ischemia was induced using the MCAO model. Under isoflurane anesthesia (1.5%–2.0% in O_2_), a silicone‐coated nylon monofilament was advanced to occlude the middle cerebral artery for 60 min, followed by reperfusion. Body temperature was maintained at 37.0°C ± 0.5°C throughout surgery. Cerebral blood flow was monitored by laser speckle contrast imaging, and only mice showing a reduction of at least 80% from baseline during occlusion were included in the analysis.

### 4.2 Isolation and Characterization of Plasma EVs

Plasma EVs were isolated via sucrose density gradient centrifugation as previously described [44]. Briefly, diluted plasma underwent sequential centrifugation (2, 000 × g and 10 000 × g) and 0.22‐µm filtration to remove debris. Crude EVs were pelleted at 110 000 × g, resuspended, and purified on a continuous sucrose gradient (0.8–2.0 m) at 100 000 × g for 16 h. Fractions with densities of 1.10–1.21 g/mL were pooled and recovered by ultracentrifugation. Characterization was performed using transmission electron microscopy (TEM), nanoparticle tracking analysis (NTA), and western blotting for ALIX (2171, Cell Signaling), CD63 (52090, Cell Signaling), TSG101(72312, Cell Signaling), and Calnexin (ab22595, Abcam) [45].

### 4.3 Production of Engineered EVs and siRNA Loading

To generate brain‐tropic EVs, HEK293T cells were cultured in serum‐free FreeStyle 293 Expression Medium (12338018, Gibco) and transfected with GNSTM‐RVG‐Lamp2b, GNSTM‐RVG‐Lamp2b‐GFP, or GNSTM‐Angiopep‐2‐Lamp2b plasmids using Lipofectamine 3000 (L3000015, Thermo Fisher Scientific) [18]. Conditioned media were harvested 48 h post‐transfection. The respective RVG‐tagged (siCircMagi1‐RVG‐EVs), GFP‐labeled RVG (siCircMagi1‐RVG‐GFP‐EVs), or Angiopep‐2‐tagged (siTrim65‐Ang2‐EVs) EVs were isolated via sequential centrifugation followed by sucrose density gradient ultracentrifugation as previously described [45].

For electroporation‐mediated cargo encapsulation, purified EVs were resuspended in Resuspension Buffer R from the Neon Transfection System and mixed at a 1:1 (w/w) ratio with specific, chemically modified siRNA mimics [46]. Both siCircMagi1 (targeting the back‐splice junction; 5'‐AAAGGACGGAGGACATTAT‐3') and siTrim65 (5'‐TGGCAGAATTATCGCAATC‐3') incorporated 2'‐O‐methyl and 2'‐fluoro modifications to ensure structural stability. To further enhance nuclease resistance and membrane permeability in vivo, these mimics were engineered with phosphorothioate linkages and a 3'‐cholesterol moiety. The dual‐modified siCircMagi1 was subsequently loaded into either siCircMagi1‐RVG‐EVs or siCircMagi1‐RVG‐GFP‐EVs. In parallel, the dual‐modified siTrim65 was encapsulated within siTrim65‐Ang2‐EVs to facilitate broad neurovascular distribution. Electroporation was performed using the Neon Transfection System at 0.5 kV with a single 10 ms pulse. Following a 30 min recovery at 37°C, unincorporated siRNA was removed by a second round of ultracentrifugation. To achieve the requisite therapeutic density of 1 × 10^11^ particles per 200 µL for systemic administration, the siRNA‐loaded EVs were further concentrated using Amicon Ultra‐15 Centrifugal Filter Units (100 kDa cutoff, Millipore). The final preparations were resuspended in sterile PBS and adjusted to the target concentration. Encapsulation efficiency and absolute siRNA copy numbers were validated by RT‐qPCR.

### 4.4 In Vivo EV Treatment Schedules and Endpoint Immune Profiling

MCAO mice were assigned to four experimental groups. Group A underwent MCAO without therapeutic EV administration. Group B received siCircMagi1‐RVG‐EVs on post‐MCAO Days 1, 5, 9, 13, 17, 21, and 25. Group C received both siCircMagi1‐RVG‐EVs and siTrim65‐Ang2‐EVs on the same seven treatment days. Group D received siCircMagi1‐RVG‐EVs on Days 1, 5, 9, 13, 17, 21, and 25, whereas siTrim65‐Ang2‐EVs were administered on Days 9, 13, 17, 21, and 25. Each EV formulation was administered by tail‐vein injection at a dose of 1 × 10^11^ particles in 200 µL sterile PBS per injection. On co‐treatment days, each formulation was administered at the stated dose. Groups C and D therefore received seven and five administrations of siTrim65‐Ang2‐EVs, respectively. Behavioral assessments were performed at the indicated time points, and histological and molecular analyses were conducted at the designated endpoints by investigators blinded to group allocation.

Blood was collected into prechilled K_2_‐EDTA‐coated tubes on post‐MCAO Day 28 and processed within 30 min to minimize ex vivo complement activation. Plasma was separated by centrifugation at 2,000 × g for 15 min at 4°C, carefully transferred without disturbing the cellular layer, aliquoted, and stored at ‐80°C until analysis. Commercial ELISA kits (Thermo Fisher Scientific) were utilized to determine plasma concentrations of IL‐6 (BMS603‐2), TNF‐α (BMS607‐3), IL‐1β (BMS6002‐2), IFN‐β (424001), C3a (EEL091), and C5a (EMHC) per the manufacturer's specified protocols. Samples were analyzed in technical duplicate, and concentrations were calculated from the corresponding standard curves.

### 4.5 Behavioral Assessments

Post‐ischemic functional recovery was evaluated using a comprehensive behavioral battery designed to assess sensorimotor integration and asymmetry [47, 48]. Global neurological deficits were quantified using mNSS, which grades motor, sensory, reflex, and balance impairments on a cumulative scale (0‐18). Motor coordination and balance were assessed via the Rotarod test, measuring the latency to fall from an accelerating rod, and the Pole test, where the total time to turn and descend a vertical apparatus was recorded to evaluate bradykinesia. Fine motor coordination was analyzed using the Grid‐walking test, calculated as the percentage of foot faults made by the contralateral forelimb relative to total steps. Spontaneous forelimb asymmetry was determined via the Cylinder test by monitoring independent wall touches of the impaired vs. unimpaired forelimb during vertical exploration. Finally, sensorimotor integration was examined using the Adhesive removal test by measuring the latency to sense and remove a tactile stimulus from the affected paw.

### 4.6 MRI and Lesion Quantification

In vivo magnetic resonance imaging was performed using a 9.4 T small‐animal MRI system (Bruker BioSpin, Ettlingen, Germany). Mice were anesthetized with 1.5%–2.0% isoflurane in an air‐oxygen mixture, and body temperature and respiratory rate were monitored throughout image acquisition. T2‐weighted images were acquired at 24 h after MCAO and at the long‐term endpoint, corresponding to experimental Days 23 and 50, respectively. Images were acquired using a two‐dimensional rapid acquisition with relaxation enhancement sequence with a repetition time of 3,500 ms, an effective echo time of 46.9 ms, a RARE factor of 8, three signal averages, a field of view of 30 × 30 mm^2^, and an acquisition matrix of 256 × 256. Thirty‐one contiguous coronal slices with a thickness of 0.5 mm and no interslice gap were acquired, providing an in‐plane resolution of 0.117 × 0.117 mm^2^ and whole‐brain coverage.

All slices covering the ischemic lesion were included in the analysis. Images were anatomically matched across animals using the lateral ventricles, striatum, and hippocampus as reference landmarks. Lesion and total brain areas were delineated on each matched slice using Bee DICOM Viewer. Lesion and total brain volumes were calculated by summing the corresponding sectional areas multiplied by slice thickness. Relative lesion burden was calculated as the lesion volume divided by the total brain volume and expressed as a percentage. Identical anatomical matching and segmentation criteria were applied to all groups by an investigator blinded to group allocation.

### 4.7 Biodistribution of Engineered EVs

To evaluate blood‐brain barrier penetration and brain‐targeting efficiency, the intrinsic fluorescence conferred by the siCircMagi1‐RVG‐GFP‐Exo platform was leveraged. MCAO mice received a systemic bolus of these dual‐functional, GFP‐labeled and siRNA‐loaded EVs via tail vein injection. Upon reaching the experimental endpoint, the mice were euthanized to facilitate the procurement of intact brains, which were immediately rinsed with heparinized saline to eliminate confounding signals from residual intravascular blood. Ex vivo optical imaging was performed using the IVIS Lumina III In Vivo Imaging System, employing excitation and emission filters precisely calibrated for GFP detection. The radiant efficiency of the localized fluorescent signal was subsequently quantified via the integrated analysis software to determine the spatial accumulation of the engineered EVs within the ischemic hemisphere.

### 4.8 TTC Staining and Infarct Volume Quantification

To evaluate ischemic injury, mice were euthanized, and their brains were rapidly removed and chilled at −20°C for 20 min to facilitate sectioning. Serial coronal slices of 2 mm thickness were prepared using a brain matrix, maintained in rostrocaudal order, and incubated in 2% 2,3,5‐triphenyltetrazolium chloride (TTC; T8877, Sigma–Aldrich) at 37°C for 15–20 min in the dark. The slices were subsequently fixed in 4% paraformaldehyde and photographed. Viable tissue stained deep red, whereas infarcted tissue remained unstained and appeared white.

All serial sections covering the ischemic territory were included in the analysis and matched across animals according to their rostrocaudal position and anatomical landmarks. For each section, the infarcted area, non‐infarcted area of the ipsilateral hemisphere, and total area of the contralateral hemisphere were delineated using ImageJ. Sectional areas were converted to volumes by multiplying each area by the section thickness and summing across all sections. Edema‐corrected infarct volume was calculated using the indirect method according to the following equation: Edema‐corrected infarct volume (%) = [(contralateral hemisphere volume—non‐infarcted ipsilateral hemisphere volume) / contralateral hemisphere volume] × 100. Identical segmentation criteria were applied to all groups by an investigator blinded to group allocation.

### 4.9 High‐Throughput RNA Sequencing

Total RNA was extracted from plasma EVs using TRIzol reagent, and RNA integrity was verified using an Agilent 2100 Bioanalyzer. To enrich for circular transcripts, total RNA was subjected to rRNA depletion followed by RNase R digestion to remove linear RNAs [49]. Sequencing libraries were constructed and sequenced on an Illumina NovaSeq 6000 platform with paired‐end reads, and these services were provided by Zhenzhi Biotechnology Co., Ltd. (Zhengzhou, China). For the computational analysis, raw reads were quality‐filtered and mapped to the human reference genome (GRCh38) using the STAR aligner. CircRNA identification was performed using a robust multiple‐algorithm cross‐validation strategy incorporating STAR, CIRCexplorer2, CIRI2, and DCC. Only circRNAs consistently detected by at least two algorithms were retained to minimize false positives. Differential expression analysis between AIS patients and healthy controls was subsequently conducted using the DESeq2 R package, with statistical significance defined by a *p‐value* < 0.05 and a |log2 fold change| > 1.

### 4.10 RAP and Mass Spectrometry

To systematically characterize the protein interactome of circMagi1, RAP coupled with mass spectrometry was performed by Biorigins Biotechnology Co., Ltd. (Shanghai, China). Biotinylated antisense probes were rationally designed to specifically hybridize with the unique BSJ sequence of circMagi1, ensuring the selective capture of the circular transcript while excluding the linear host mRNA. In brief, cell lysates were incubated with these circMagi1‐specific probes or control probes, and the resulting RNA‐protein complexes were captured using streptavidin‐coated magnetic beads. The enriched proteins were eluted, resolved by SDS‐PAGE, and visualized via silver staining to assess sample quality. Protein identities were subsequently determined using LC‐MS/MS, and candidate interactors were filtered based on spectral counts and enrichment fold change relative to the control group.

### 4.11 RIP Assay

To systematically identify the repertoire of circular RNAs interacting with Anxa2, RIP assays were conducted using the Magna RIP RNA‐Binding Protein Immunoprecipitation Kit (17‐700, Millipore). Endothelial cell lysates were prepared and incubated with magnetic beads conjugated to either a specific anti‐Anxa2 antibody or a negative control IgG overnight at 4°C with rotation. Following stringent washing to remove non‐specific binding, protein‐RNA complexes were treated with Proteinase K, and the co‐precipitated RNA was extracted via phenol‐chloroform purification. To characterize the Anxa2‐bound circRNA landscape, the eluted RNA was subjected to library preparation and high‐throughput circRNA sequencing analysis by Novogene Co., Ltd. (Beijing, China). Subsequently, to specifically validate the interaction with the target candidate, the enrichment of circMagi1 in the Anxa2‐bound fraction was quantified via absolute real‐time qPCR and normalized to the input fraction.

### 4.12 Quantitative Real‐Time PCR Analysis of circMagi1

Total RNA was extracted from purified EVs or tissues using TRIzol reagent. To ensure rigorous normalization independent of linear housekeeping gene variability, a defined amount of exogenous circular RNA spike‐in (synthesized by GenScript, Nanjing, China) was introduced into each sample lysate prior to extraction. This spike‐in control was generated in vitro from a Lambda phage sequence (unaligned to the human or mouse genome) to serve as a reliable external reference. Reverse transcription was performed using the SuperScript IV First‐Strand Synthesis System (Thermo Fisher Scientific) with random hexamers. Quantitative PCR was subsequently conducted using SYBR Green Master Mix on a LightCycler 480 II System (Roche, Rotkreuz, Switzerland). Crucially, divergent primers were specifically designed to span the back‐splice junction of circMagi1, ensuring the exclusive amplification of the circular isoform. Relative expression levels were calculated using the 2^−△△Ct^ method, normalized to the Ct values of the exogenous circular spike‐in.

### 4.13 Absolute Quantitative Real‐Time PCR Analysis of siRNA

Absolute copy numbers of therapeutic siCircMagi1 and siTrim65 in engineered EV preparations and brain tissue were determined using sequence‐matched synthetic siRNA oligonucleotides (GenePharma Co., Ltd., Suzhou, China) as quantitative standards. Seven‐point standard curves were generated for each target using 10‐fold serial dilutions starting at 1 µM. Total RNA was extracted from freshly collected brain tissue or purified EV pellets using the miRNeasy Mini Kit (217004, QIAGEN). RNA concentration and purity were assessed using a NanoDrop 2000 spectrophotometer (Thermo Fisher Scientific, Waltham, MA, USA), with purity evaluated from the A260/A280 ratio.

For cDNA synthesis, reverse transcription was executed using the miRNA First‐Strand Synthesis Kit (638313, Takara) in accordance with the manufacturer's protocol. This procedure facilitated the 3'‐polyadenylation of the small RNA species followed by cDNA synthesis using a universal oligo‐dT adapter primer to ensure high specificity for the target siRNAs. Quantitative PCR was performed using SYBR Green PCR Master Mix (SR1120, Solarbio) on a Rotor‐Gene Q real‐time PCR cycler. The thermal cycling conditions consisted of an initial denaturation at 95°C for 3 min, followed by 40 cycles of denaturation at 95°C for 10 s and combined annealing/extension at 60°C for 30 s. The absolute copy numbers in experimental samples were interpolated from the respective standard curves as previously described, yielding high linearity with coefficient of determination (R^2^) values of 0.9926 for siCircMagi1 and 0.9898 for siTrim65. All reactions were conducted in triplicate to maintain technical reproducibility. Final data were normalized to the initial sample input, with concentrations expressed as copies per mg for brain tissues and copies per 10^10^ particles for EV‐packaged preparations.

### 4.14 Isolation and Culture of Primary Neurons and Brain Microvascular Endothelial Cells

Primary neurons and brain microvascular endothelial cells were isolated from both embryonic and adult C57BL/6 mice using magnetic activated cell sorting technology to ensure high purity and consistency [50, 51]. Whole brain tissues were dissected and meninges along with large blood vessels were carefully removed. Single‐cell suspensions were prepared using the Neural Tissue Dissociation Kit (130‐093‐231, Miltenyi Biotec) for embryos and the Adult Brain Dissociation Kit (130‐107‐677, Miltenyi Biotec) for adult mice according to the manufacturer's instructions. A negative selection strategy was employed to deplete non‐neuronal lineages using the Neuron Isolation Kit (130‐115‐389, Miltenyi Biotec). This involved sequential incubation with biotinylated antibodies and magnetic microbeads targeting non‐neuronal cells. The unlabeled neurons were collected in the flow‐through fraction. To validate the isolation efficiency, aliquots of the sorted cells were fixed, permeabilized, and immunostained with anti‐NeuN antibodies (MA5‐33103, Thermo Fisher Scientific) followed by flow cytometric analysis to assess the purity of the neuronal population. The purified neurons were seeded onto plates coated with Poly‐D‐lysine (P6407, Sigma) and cultured in Neurobasal Medium (21103049, Gibco) supplemented with B‐27 Supplement (17504044, Gibco) and GlutaMAX (35050061, Gibco). Adult neurons were maintained in Neurobasal‐A Medium (10888022, Gibco) to support long‐term survival.

A positive selection strategy was employed to isolate primary BMECs from whole brain tissues of both embryonic and adult mice [50]. Tissues from both developmental stages were harvested and subjected to enzymatic digestion to obtain single‐cell suspensions. For adult brain tissues, the cell suspension was specifically subjected to BSA density gradient centrifugation to remove the abundant myelin debris, whereas this step was omitted for embryonic tissues. Subsequently, the single‐cell suspensions from both sources were incubated with CD31 (PECAM‐1) MicroBeads (130‐097‐418, Miltenyi Biotec) and passed through a magnetic separation column. The magnetically enriched CD31 positive endothelial cells were eluted after stringent washing to remove unbound cells. The purity of the enriched population was verified by flow cytometry using fluorophore‐conjugated anti‐CD31 antibodies (ab7388, Abcam). The purified cells were subsequently cultured in Endothelial Cell Medium (1001, ScienCell) on dishes coated with Bovine Plasma Fibronectin (8248, ScienCell) and used at passage 1 or 2.

### 4.15 Oxygen‐Glucose Deprivation/Reoxygenation Model

To simulate ischemic injury in vitro, primary neurons and BMECs were subjected to oxygen‐glucose deprivation (OGD) followed by reoxygenation. The culture medium was replaced with deoxygenated glucose‐free DMEM (11966025, Gibco) and the cells were placed in a hypoxia chamber containing a gas mixture of 1% O_2_, 5% CO_2_, and 94% N_2_ at 37°C. After 2 h of hypoxia, the cells were returned to normoxic conditions. The medium was replaced with fresh culture medium to initiate reoxygenation for 24 h.

To investigate the regulatory effects of neuronal signals on brain microvascular endothelial cells, a non‐contact co‐culture system was established using Transwell inserts with a 0.4 µm pore size polyester membrane (3401, Corning). In this configuration, primary neurons were seeded on the apical side (upper chamber) of the insert while BMECs were cultured in the basolateral compartment (lower chamber). To ensure the survival of both cell types during the co‐culture period, the medium in both chambers was replaced with a co‐culture medium consisting of a 1:1 mixture of complete Neurobasal medium and Endothelial Cell Medium. To determine whether the vascular regulation was mediated by EVs, the EV release inhibitor GW4869 (D1692, Sigma–Aldrich) was introduced. Specifically, the neurons serving as donor cells were pre‐treated with 10 µM GW4869 for 12 h prior to the OGD procedure. During the subsequent 2‐hour OGD phase, GW4869 was maintained in the glucose‐free medium to continuously block EV secretion. The effects of the co‐culture on endothelial function were analyzed following the reoxygenation period.

### 4.16 Electrophoretic Mobility Shift Assay (EMSA)

To validate the direct interaction between Anxa2 and the target RNA sequence, an electrophoretic mobility shift assay was performed using the LightShift Chemiluminescent RNA EMSA Kit (20158, Thermo Fisher Scientific). Biotin‐labeled RNA oligonucleotides corresponding to the wild‐type binding motif of circMagi1 and a mutant sequence were synthesized by GenScript. The recombinant GST‐tagged Anxa2 protein was purified and incubated with the biotinylated probes in a binding buffer supplemented with glycerol, KCl, and tRNA to prevent non‐specific binding. The reaction was allowed to proceed for 20 min at room temperature. To demonstrate the specificity of the interaction, a 100‐fold molar excess of unlabeled wild‐type probe (cold competitor) was added to the reaction mixture to compete for binding sites. For the supershift assay, an anti‐Anxa2 antibody was added to the reaction and incubated for an additional 20 min to identify the protein component within the complex. A biotin‐labeled mutant probe was also used to verify sequence‐specific binding. The reaction mixtures were resolved on a 6% non‐denaturing polyacrylamide gel in TBE buffer and subsequently transferred onto a positively charged nylon membrane. Following UV cross‐linking, the biotin‐labeled RNA signals were detected using a streptavidin‐horseradish peroxidase conjugate and a chemiluminescent substrate.

### 4.17 In Silico Structural Modeling of the Protein‐RNA Complex

The structural basis of the interaction between circMagi1 and ANXA2 was investigated using the AlphaFold 3 server, which employs a diffusion‐based generative architecture for high‐accuracy modeling of biomolecular complexes [52]. The full‐length amino acid sequence of ANXA2 and the nucleotide sequence of the circMagi1 binding motif were input simultaneously to generate the joint three‐dimensional model. Evaluation of the predicted interaction interface relied on the Predicted Aligned Error (PAE) matrix, where low PAE values between the protein and RNA chains indicated a high‐confidence relative alignment. Furthermore, the interface predicted Template Modeling (ipTM) score served as a primary metric to assess the overall quality of the complex structure. The top‐ranked model exhibiting the highest confidence score was selected for detailed structural analysis. Visualization of the complex, including the identification of key interacting residues, hydrogen bonds, and electrostatic surface potentials, was performed using PyMOL (Schrödinger, LLC).

### 4.18 Ubiquitination Assay

Human Umbilical Vein Endothelial Cells (HUVECs) were seeded in 6‐well plates and cotransfected with plasmids encoding HA‐tagged ubiquitin (HA‐Ub) along with the indicated shRNA or overexpression vectors using Lipofectamine 3000 (L3000015, Thermo Fisher Scientific). Forty‐eight hours after transfection, the cells were treated with 10 µM MG132 (M7449, Sigma) for 6 h. Whole‐cell lysates were subsequently prepared using a modified RIPA lysis buffer supplemented with a protease inhibitor cocktail and N‐ethylmaleimide. A portion of the lysate was reserved as input to verify protein expression levels. The remaining lysates were immunoprecipitated with an anti‐Anxa2 antibody and Protein A/G Magnetic Beads (88803, Thermo Fisher Scientific) at 4°C overnight. The immunoprecipitated protein complexes were washed stringently to remove non‐specific binding, eluted by boiling in SDS loading buffer, and resolved by SDS‐PAGE. The polyubiquitination signal was detected via western blotting using an anti‐HA antibody. Concurrently, the Input fractions were analyzed using anti‐Trim65 and anti‐GAPDH antibodies to validate transfection efficiency and loading consistency.

### 4.19 RNA‐Dependent ANXA2‐TRIM65 Recruitment and Cell‐Free Ubiquitination Assays

To examine whether intact circMagi1 enhances ANXA2‐TRIM65 engagement, RNase rescue co‐immunoprecipitation, purified recruitment, and cell‐free ubiquitination assays were performed. For RNase rescue Co‐IP [53], HUVEC lysates were treated with RNase A/T1 (EN0551, Thermo Fisher Scientific) for RNA digestion. After RNase removal or inactivation, purified WT circMagi1, circMagi1_Mut, or scrambled circRNA was added back to the lysates. ANXA2 was immunoprecipitated using anti‐ANXA2 antibody (ab321903, Abcam), followed by immunoblotting for TRIM65 (MA5‐55752, Invitrogen) and ANXA2. IgG immunoprecipitation was used as a negative control. TRIM65 co‐immunoprecipitation was quantified after normalization to immunoprecipitated ANXA2.

For the purified recruitment assay, immobilized ANXA2 beads were incubated with purified TRIM65 in the presence or absence of WT circMagi1, circMagi1_Mut, scrambled circRNA, or RNase A/T1 treatment. Control beads and no‐TRIM65 reactions were included as negative controls. After washing, bead‐bound proteins were analyzed by immunoblotting for TRIM65 and ANXA2. TRIM65 recruitment was quantified as the amount of bead‐bound TRIM65 normalized to bead‐bound ANXA2.

For the cell‐free ubiquitination assay [54], purified ANXA2 was incubated with TRIM65, E1 (E‐305, R&D systems), E2 (E2‐616, R&D systems), ATP, and HA‐ubiquitin (U‐110, R&D systems) or K48‐only ubiquitin (UM‐K480, R&D systems) in the presence of WT circMagi1, circMagi1_Mut, scrambled circRNA, or RNase A/T1 treatment. Reactions lacking ATP or TRIM65 were used as negative controls. After incubation at 37°C for 60 min, the reaction was terminated by adding IP lysis buffer containing protease inhibitors and N‐ethylmaleimide. ANXA2 was immunoprecipitated and analyzed by immunoblotting for HA‐ubiquitin/K48‐ubiquitin and ANXA2. Ubiquitinated ANXA2 signals were quantified from the high‐molecular‐weight smear and normalized to total immunoprecipitated ANXA2.

### 4.20 Neuronal EV Reporter Tracing and Endothelial‐Cell Analysis

To trace neuron‐derived vesicular signals in vivo, AAV‐hSyn‐CD63‐mCherry was administered by intracerebroventricular injection at 2 µL per lateral ventricle (1 × 10^12^ vg/mL) per mouse. After a 3‐week expression period, mice were subjected to MCAO, and peri‐infarct brain tissue was collected 24 h after ischemic injury. CD63‐mCherry was used as a neuronal CD63‐associated vesicular reporter and was not interpreted as direct labeling of endogenous circMagi1.

For tissue imaging, circMagi1 fluorescence in situ hybridization was performed using probes spanning the back‐splice junction, followed by immunofluorescence staining for neuronal and endothelial markers. Confocal images were acquired from peri‐infarct regions using identical acquisition settings across groups. CD31‐positive endothelial regions were segmented, and overlapping circMagi1 FISH puncta and neuronal CD63‐mCherry puncta within CD31‐positive areas were quantified. Overlap was reported as overlap spot density per CD31‐positive area.

For flow cytometry, peri‐infarct brain tissue was enzymatically dissociated into single‐cell suspensions. Live single cells were gated, and CD31‐positive endothelial cells were analyzed for neuronal CD63‐mCherry fluorescence. Where indicated, cell suspensions were subjected to acid‐wash surface stripping before flow cytometry to reduce surface‐associated vesicular signals. Mean fluorescence intensity was normalized to the sham group. For sorted‐cell RT‐qPCR, CD31‐positive endothelial cells were collected, supplemented with an exogenous circular RNA spike‐in before RNA extraction, and analyzed using divergent primers spanning the circMagi1 back‐splice junction.

### 4.21 Tube Formation Assay

To evaluate the in vitro angiogenic capability, tube formation assays were performed using HUVECs subjected to the indicated treatments. 48‐well plates were pre‐coated with 100 µL of Growth Factor Reduced Matrigel (356230, Corning) per well and incubated at 37°C for 30 min to allow polymerization. The pre‐treated cells were harvested, resuspended in endothelial cell medium, and seeded onto the solidified Matrigel matrix at a density of 6 × 10^4^ cells per well. The plates were incubated at 37°C in a 5% CO_2_ humidified atmosphere. After 4 to 6 h, the formation of capillary‐like tubular structures was examined and photographed using an inverted microscope. Three randomly selected fields per well were captured. The angiogenic ability was quantified by measuring the total tube length and the number of junctions using the angiogenesis analyzer plugin for ImageJ software.

### 4.22 Transwell Invasion Assay

Transwell invasion assays were performed using 24‐well Transwell inserts with 8.0 µm pore size polycarbonate membranes (3422, Corning). The upper surface of the membrane was pre‐coated with Growth Factor Reduced Matrigel (356230, Corning) diluted 1: 8 in serum‐free medium to simulate the extracellular matrix barrier. HUVECs subjected to the indicated treatments were harvested and resuspended in serum‐free endothelial cell medium at a density of 5 × 10^4^ cells per insert. The cell suspension was added to the upper chamber, while 600 µL of complete medium containing 10% FBS was placed in the lower chamber to serve as a chemoattractant. Following incubation at 37°C for 24 h, the non‐invading cells remaining on the upper surface were gently removed with a cotton swab. The cells that had invaded through the matrigel and adhered to the lower surface of the membrane were fixed with 4% paraformaldehyde for 15 min and stained with 0.1% Crystal Violet for 10 min. The invaded cells were imaged and counted in five randomly selected fields per sample using an inverted microscope.

### 4.23 Immunofluorescence Staining

Immunofluorescence staining was performed on paraffin‐embedded brain tissue sections to visualize the cellular localization of target proteins and their association with specific cell types. Brain tissues were fixed in 4% paraformaldehyde, embedded in paraffin, and sectioned at a thickness of 4 µm. The sections were deparaffinized in xylene, rehydrated through a graded ethanol series, and subjected to antigen retrieval in citrate buffer (pH 6.0) via microwave heating. After blocking with 5% Bovine Serum Albumin for 1 h at room temperature, the sections were incubated overnight at 4°C with specific primary antibodies targeting Anxa2 (RM1229, Abcam), Trim65 (orb1721077, Biorbyt), CD31 (ab7388, Abcam), IBA1 (ab283319, Abcam), and CD68 (ab283654, Abcam) [55]. Following three washes with PBS, the sections were incubated with the corresponding Alexa Fluor 488‐ or 594‐conjugated secondary antibodies for 1 h at room temperature in the dark. Nuclei were counterstained with DAPI. Fluorescence images were captured using a confocal laser scanning microscope.

### 4.24 scRNA‐seq and Data Analysis

scRNA‐seq library construction and sequencing were performed by Biorigins Biotechnology Co., Ltd. (Zhengzhou, China) using the 10 × Genomics platform. Raw sequencing data were processed using Cell Ranger for demultiplexing and alignment to the reference genome to generate the gene‐barcode matrix. Downstream analysis was conducted using the Seurat R package. Following quality control to remove low‐quality cells and potential doublets, the data were normalized, scaled, and subjected to Principal Component Analysis for dimensionality reduction. Unsupervised clustering was performed, and the cell clusters were visualized using Uniform Manifold Approximation and Projection. Cell types were annotated based on the expression of canonical marker genes, and the relative cell type proportions were quantified across different groups.

Endothelial cells were computationally subsetted from the total cell population for downstream re‐clustering and differential expression analysis. Identification of Differentially Expressed Genes (DEGs) between groups was performed using the Wilcoxon rank‐sum test, with significance thresholds set at |log_2_FC| > 1 and *p‐value* < 0.05. Gene Ontology (GO) enrichment and Kyoto Encyclopedia of Genes and Genomes (KEGG) pathway analyses were conducted using Metascape to elucidate the biological functions and signaling pathways associated with the DEGs.

Single‐cell trajectory analysis of the endothelial sub‐clusters was performed using Monocle 2 [46]. Ordering genes were selected based on differential expression and dispersion (*q*‐value < 0.1). The dimensional reduction was carried out using the DDRTree method to reconstruct the differentiation trajectory. The pseudotime axis was oriented to model the transition from *Vegfa^low^* to *Vegfa^high^* states, reflecting the dynamic changes in endothelial cell phenotypes.

### 4.25 Key Reagents and Resources

Supplier and catalog information for the antibodies, assay kits, recombinant proteins, enzymes, inhibitors, and other key reagents used in this study is provided in Table S7.

### 4.26 Quantification and Statistical Analysis

Statistical analyses were performed using GraphPad Prism version 9.0. Data are presented as mean ± s.d. unless otherwise indicated. The number of biological replicates and statistical tests used are specified in the corresponding figure legends. Data distributions were assessed using the Shapiro–Wilk test. Comparisons between two independent groups were performed using unpaired two‐tailed Student's t‐tests, whereas matched observations were analyzed using paired two‐tailed Student's t‐tests. Comparisons among three or more groups were performed using one‐way analysis of variance (ANOVA) followed by Tukey's multiple‐comparisons test. Experiments involving two factors were analyzed using two‐way ANOVA followed by Tukey's or Šídák's multiple‐comparisons test, as specified in the corresponding figure legends.

Longitudinal behavioral outcomes were analyzed using two‐way repeated‐measures ANOVA, with treatment as the between‐subject factor and time as the within‐subject factor. Treatment × time interactions were used to determine whether behavioral changes over time differed among treatment groups. Comparisons at prespecified endpoints were analyzed separately using one‐way ANOVA followed by Tukey's multiple‐comparisons test. For each group, the residual behavioral fraction was calculated as the mean Day 28 value divided by the mean Day 1 value. The descriptive advantage of Group D over Group B was calculated as ∆ = [(X_B, 28_/X_B, 1_)‐(X_D, 28_/X_D, 1_)] × 100. Positive Δ values therefore indicate a lower residual behavioral deficit in Group D.

Image acquisition and quantitative analyses were performed using consistent acquisition settings, thresholding, and segmentation criteria by investigators blinded to group allocation. All statistical tests were two‐sided where applicable, and p < 0.05 was considered statistically significant. Statistical significance is denoted as ^*^ *p <* 0.05, ^**^ *p <* 0.01, and ^***^ *p <* 0.001. Exact *P‐value*s are reported where appropriate.

## Author Contributions

G.J., Y.B., H.W., X.G., and Y.X. conceived the project and supervised the overall study. Y.B., Q.L., and B.Z. led project execution and performed the molecular mechanism studies. Y.B. and Q.L. focused on the engineering, large‐scale purification, and physicochemical characterization of the dual‐targeting EVs. Y.B., X.Q., and S.T. isolated the primary neurons and brain microvascular endothelial cells. Y.B., B.Z., W.W., and C.Z. conducted the comprehensive bioinformatic analyses, including high‐throughput sequencing interpretation, single‐cell transcriptomic profiling, and AlphaFold 3‐based structural modeling. L.G. and X.Q. performed the qPCR assays. X.G., R.G., and C.M. provided clinical insights and contributed to the pathological assessment. Q.L. and N.H. established the rodent stroke models and performed the behavioral assessments. Y.B. drafted the manuscript. Y.B., G.J., J.C., H.W., X.G., and Y.X. reviewed and revised the manuscript. All authors discussed the results and approved the final manuscript.

## Conflicts of Interest

The authors declare no conflicts of interest.

## Supporting information




**Supporting File 1**: advs77808‐sup‐0001‐SuppMat.pdf.

**Supporting File 2**: advs77808‐sup‐0002‐TableS1.xlsx.

**Supporting File 3**: advs77808‐sup‐0003‐TableS2.xlsx.

**Supporting File 4**: advs77808‐sup‐0004‐TableS3.xlsx.

**Supporting File 5**: advs77808‐sup‐0005‐TableS4.xlsx.

**Supporting File 6**: advs77808‐sup‐0006‐TableS5.xlsx.

**Supporting File 7**: advs77808‐sup‐0007‐TableS6.xlsx.

**Supporting File 8**: advs77808‐sup‐0008‐TableS7.xlsx.

## Data Availability

The main data supporting the findings of this study are available within the Article and its Supplementary Information. The processed multi‐omics datasets (including single‐cell RNA‐seq expression matrices, high‐throughput circRNA sequencing quantification, and RAP/RIP analysis results) and the AlphaFold 3‐predicted structural models generated in this study have been deposited in Mendeley Data under doi: 10.17632/hb7tvkkc3b.2. All other data are available from the corresponding authors upon reasonable request.
