# Supplementary material for: Neuron‐Derived Vesicular circMagi1 Restrains Vascular Repair After Ischemic Stroke by Promoting TRIM65‐Mediated ANXA2 Degradation
Source: Adv Sci (Weinh). 2026 Oct 15:e77808. Online ahead of print. doi: 10.1002/advs.77808 (PMC13673058; doi:10.1002/advs.77808)
Supplement: Supplementary file 1 [file ADVS-9999-e77808-s002.pdf]

Supplementary information

Neuron-derived vesicular circMagi1 restrains vascular repair after ischemic stroke by promoting TRIM65-mediated ANXA2 degradation

Supplementary Figures

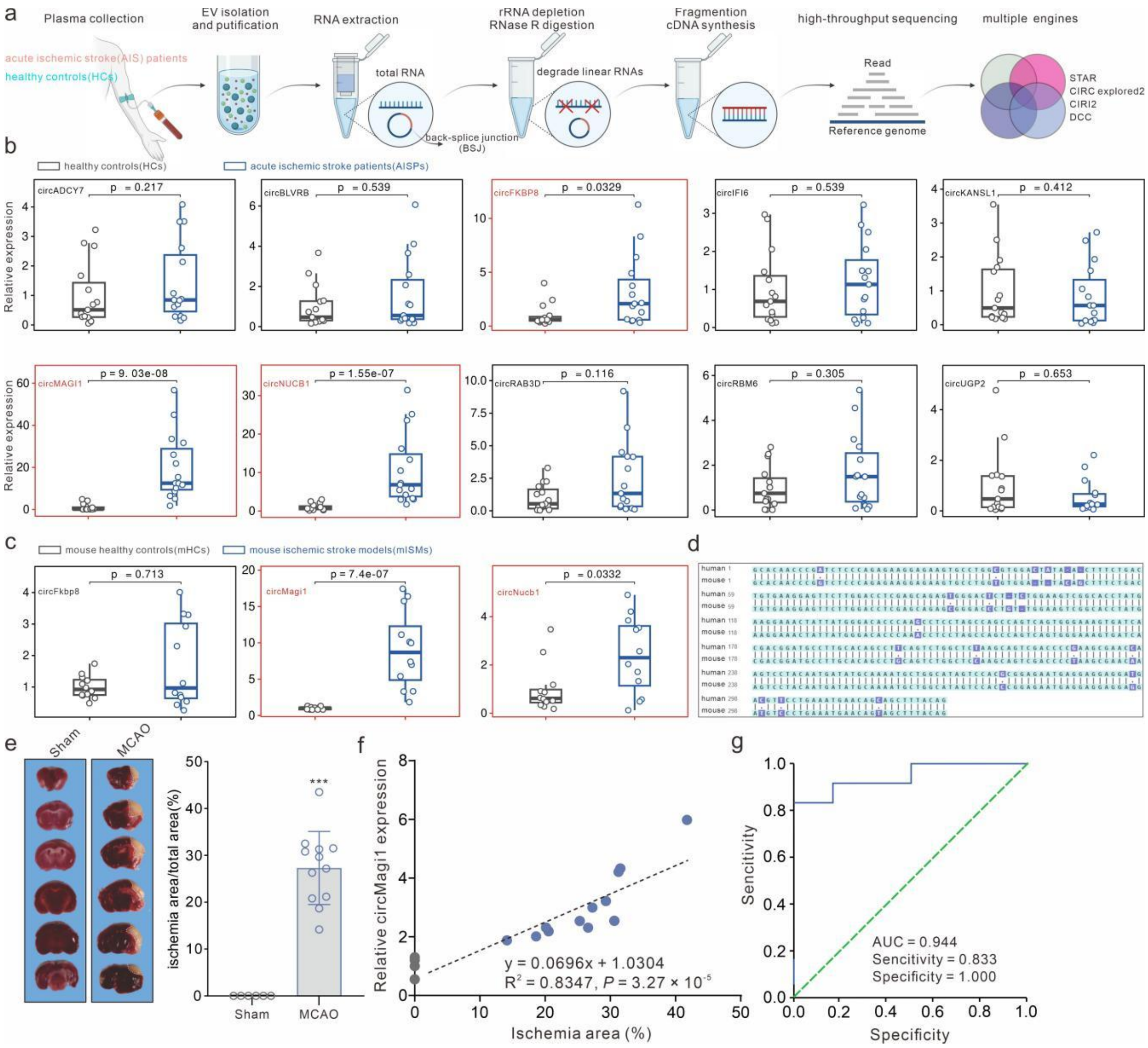

Fig. S1: Validation of circulating EV-packaged circMagi1 in human and mouse ischemic stroke. Related to Fig. 1.

(a) Workflow for plasma EV isolation, RNA extraction, RNase R enrichment, library preparation, high-throughput sequencing, and circRNA identification using multiple detection algorithms. (b) RT-qPCR validation of candidate EV-packaged circRNAs in plasma from patients with acute ischemic stroke and healthy controls. (c) RT-qPCR validation of conserved candidate circRNAs in plasma EVs from MCAO mice and healthy control mice. (d) Sequence alignment showing the conservation of circMagi1 between humans and mice. (e) Representative TTC-stained brain sections and quantification of ischemic area in sham and MCAO mice. (f) Association between plasma EV-packaged circMagi1 abundance and ischemic area across the analyzed mouse cohort. The solid line represents the fitted simple linear regression. (g) Receiver operating characteristic curve evaluating the ability of plasma EV-packaged circMagi1 to distinguish MCAO mice from controls. Data are presented as mean ± s.d. or as box-and-whisker plots, as indicated. RT-qPCR comparisons in (b,c) were performed on ΔCt values using two-sided Welch's t-tests, with Benjamini-Hochberg correction applied separately within the

human and mouse candidate sets. Adjusted-*P* values are shown in the corresponding panels. Infarct burden in (e) was compared using an exact two-sided Mann-Whitney test. The association in (f) was evaluated using simple linear regression; the coefficient of determination (R^2) and regression *P*-value are shown in the panel. ROC performance in (g) is reported as the area under the curve, sensitivity, and specificity. $^{***}P < 0.001$.

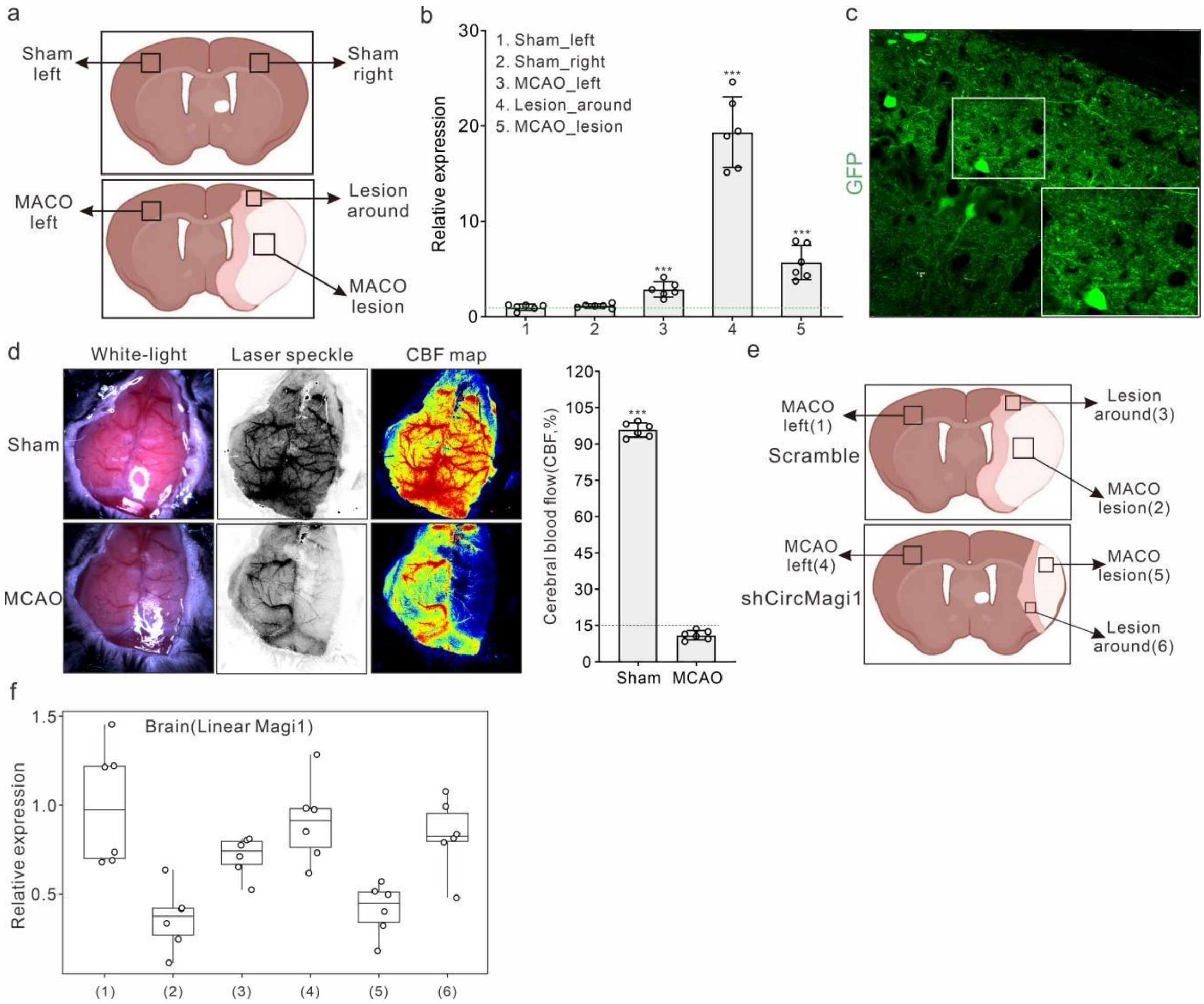

Fig. S2: Regional induction of circMagi1 and validation of AAV-mediated silencing specificity. Related to Fig. 2.

(a) Schematic definition of sampled regions in sham and MCAO brains. (b) RT-qPCR analysis of circMagi1 expression in the indicated brain regions, showing preferential induction in peri-lesional tissue after MCAO. (c) Representative GFP fluorescence confirming AAV expression in brain tissue after intracerebroventricular delivery. (d) Laser speckle contrast imaging showing cerebral blood flow reduction after MCAO induction, with quantification of relative CBF. (e) Schematic definition of sampled regions in scramble and AAV-shCircMagi1-treated MCAO mice. (f) RT-qPCR analysis of linear *Magi1* mRNA in the indicated brain regions, showing that AAV-shCircMagi1 did not suppress the corresponding linear transcript. Data are presented as mean ± s.d. or box-and-whisker plots as shown. Regional expression data were analyzed using a linear mixed-effects model with brain region as a fixed effect and mouse as a random intercept. Treatment-dependent regional effects were evaluated using a mixed-design two-way ANOVA including treatment, region, and treatment × region interaction, followed by Šídák-adjusted comparisons between corresponding regions. *p*-values are indicated as $^{***}P < 0.001$.

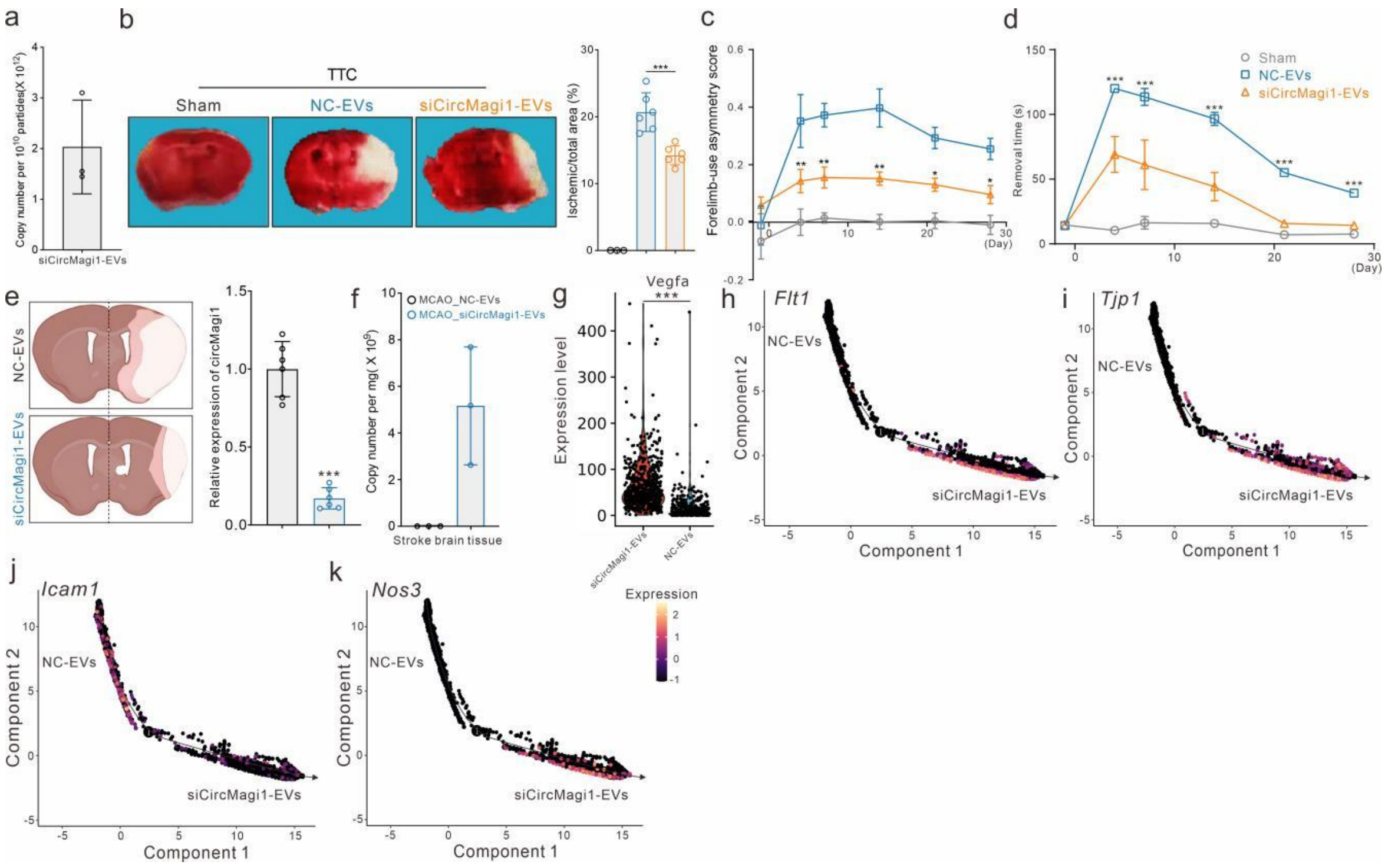

Fig. S3: Validation of RVG-EV-mediated siCircMagi1 delivery and additional therapeutic analyses. Related to Fig. 3.

(a) Absolute quantification of siCircMagi1 loading in RVG-EVs, expressed as copy number per 10^{10} EV particles (n = 3 independent EV preparations). (b) Representative TTC-stained brain sections obtained on post-MCAO Day 7 and quantification of infarct area relative to total brain area. Sham, n = 3 mice; NC-EVs and siCircMagi1-RVG-EVs groups, n = 6 mice per group. (c,d) Longitudinal assessment of forelimb-use asymmetry scores in the cylinder test (c) and adhesive-removal time (d) in sham, NC-EV-treated, and siCircMagi1-RVG-EVs-treated mice (n = 6 mice per group). (e) Schematic definition of the analyzed ischemic brain region and RT-qPCR validation of circMagi1 knockdown following siCircMagi1-RVG-EVs administration (n = 6 mice per group). (f) Absolute quantification of siCircMagi1 abundance in ischemic brain tissue (n = 3 mice per group). (g) Single-cell analysis of Vegfa expression in endothelial cells from the NC-EVs and siCircMagi1-RVG-EVs groups. The single-cell RNA-sequencing dataset comprised three biological replicates per group. (h-k) Pseudotime feature plots showing the expression dynamics of Flt1 (h), Tjp1 (i), Icam1 (j), and Nos3 (k) during endothelial-state transition. Data are presented as mean ± s.d. Infarct-area measurements in (b) were analyzed using one-way ANOVA followed by Tukey's multiple-comparisons test. Longitudinal behavioral outcomes in (c,d) were analyzed using two-way repeated-measures ANOVA, including treatment, time, and the treatment × time interaction, followed by Tukey's multiple-comparisons test. RT-qPCR data in (e) were analyzed using an unpaired two-tailed Student's t-test. Absolute siCircMagi1 measurements in (f) are presented descriptively because the signal was undetectable in the NC-EVs control group. Single-cell differential expression in (g) was assessed using the Wilcoxon rank-sum test with Benjamini-Hochberg correction. Panels (h-k) provide descriptive visualization of gene-expression dynamics along pseudotime. $^{*}P < 0.05$, $^{**}P < 0.01$, and $^{***}P < 0.001$.

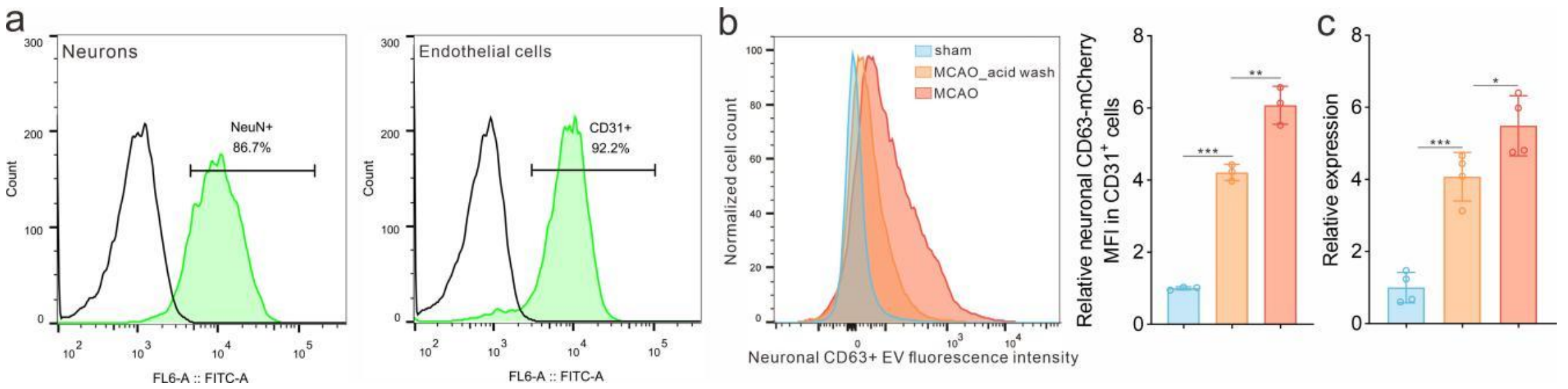

Fig. S4: Detection of neuronal CD63-mCherry reporter and circMagi1 signals in sorted endothelial cells. Related to Fig. 4.

(a) Representative flow-cytometry profiles confirming the purity of the isolated neuronal and endothelial fractions, with representative preparations containing 86.7% NeuN$^+$ neurons and 92.2% CD31$^+$ endothelial cells. (b) Representative flow-cytometry distributions of neuronal CD63-mCherry reporter fluorescence in sorted CD31$^+$ endothelial cells from sham mice, untreated MCAO mice, and MCAO mice subjected to ex vivo acid-wash surface stripping. The accompanying graph shows neuronal CD63-mCherry mean fluorescence intensity in the CD31$^+$ endothelial fraction relative to the sham group (n = 3 mice per group). (c) RT-qPCR quantification of circMagi1 in sorted CD31$^+$ endothelial cells using back-splice-junction-spanning divergent primers and normalization to an exogenous circular RNA spike-in (n = 3 mice per group). Data are presented as mean ± s.d. Statistical significance was determined using one-way ANOVA followed by Tukey's multiple-comparison test. Significance is denoted by $^{*}P < 0.05$, $^{**}P < 0.01$, and $^{***}P < 0.001$.

.

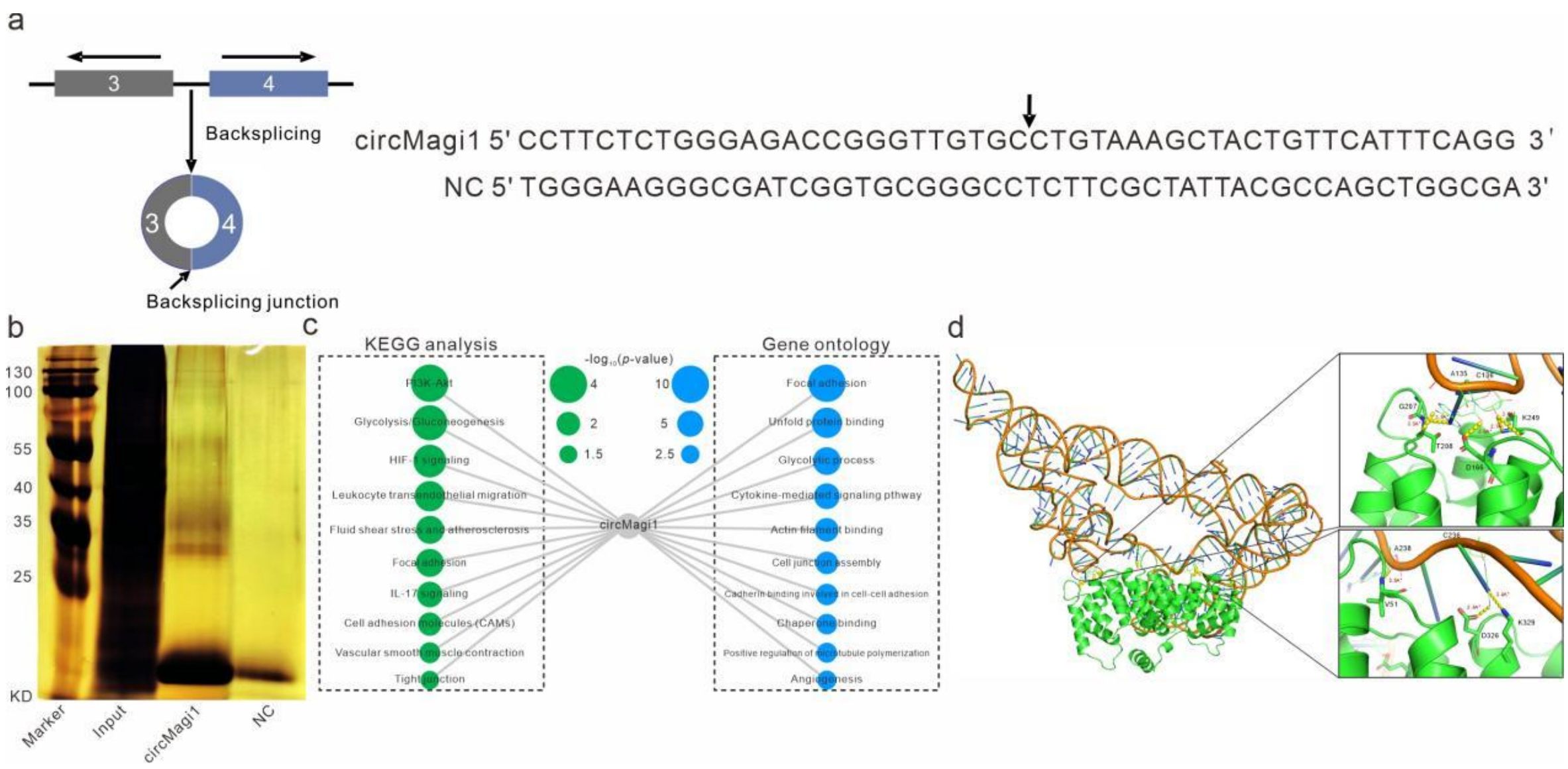

Fig. S5: RAP-MS identification and structural modeling of circMagi1-associated proteins. Related to Fig. 5.

(a) Design of the biotinylated circMagi1 probe targeting the back-splice junction and the corresponding negative control probe. (b) Silver-stained SDS-PAGE gel showing proteins retrieved by circMagi1 RAP compared with the negative control. (c) KEGG pathway and Gene Ontology enrichment analyses of circMagi1-associated proteins identified by RAP-MS. Dot size represents gene number, and color indicates enrichment significance. (d) Additional AlphaFold-based structural modeling of the circMagi1-ANXA2 complex, highlighting predicted auxiliary interaction interfaces.

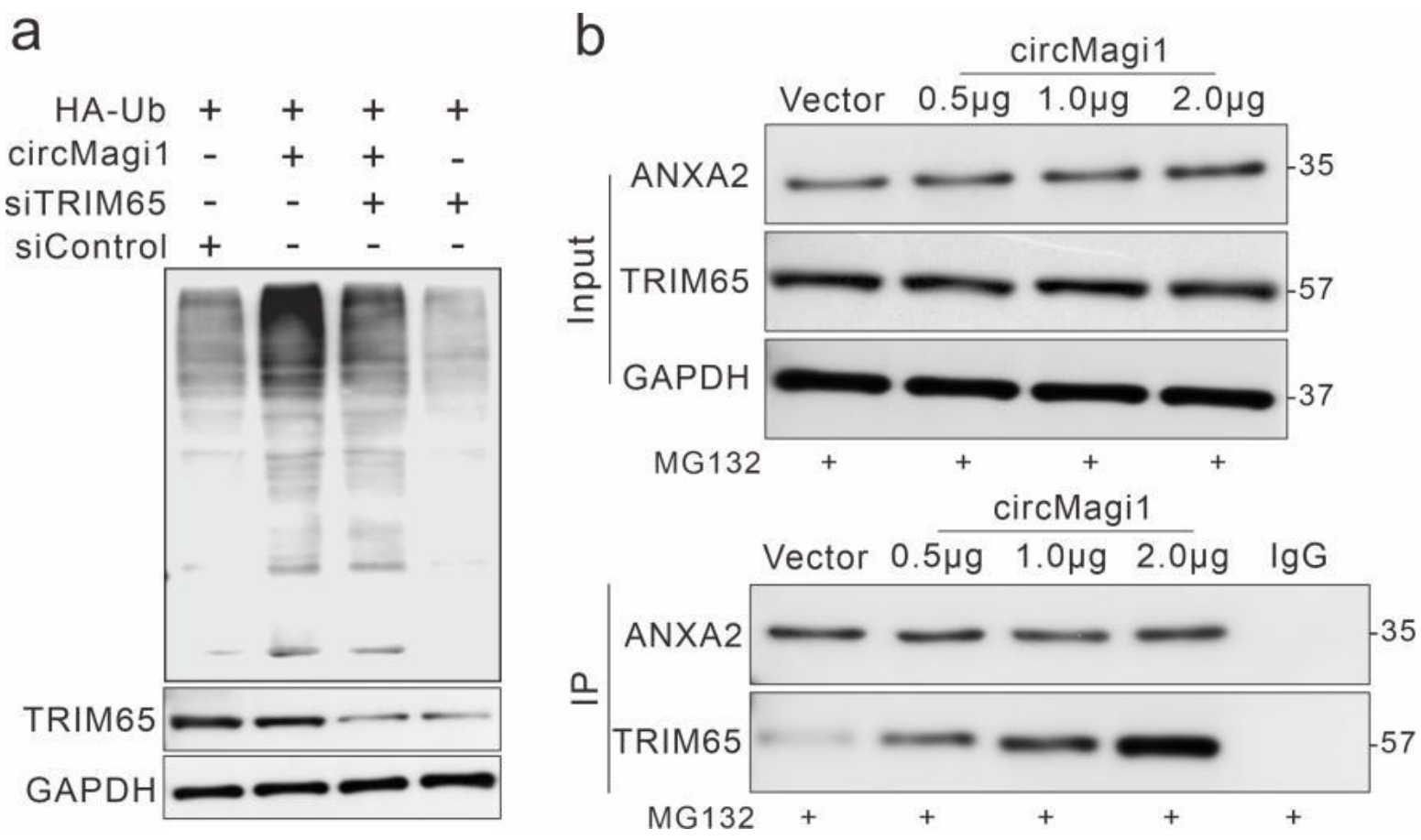

Fig. S6: CircMagi1 enhances TRIM65-dependent ANXA2 ubiquitination and ANXA2-TRIM65 association in cells. Related to Fig. 6.

(a) Cell-based ubiquitination assay in HUVECs expressing HA-ubiquitin with circMagi1 and/or siTRIM65. ANXA2 was immunoprecipitated and immunoblotted for HA-ubiquitin to detect ubiquitinated ANXA2 species. TRIM65 and GAPDH immunoblots confirm knockdown efficiency and loading. (b) Dose-dependent Co-IP analysis in MG132-treated HUVECs showing that increasing circMagi1 expression enhances the amount of TRIM65 recovered with ANXA2. IgG served as the negative immunoprecipitation control.

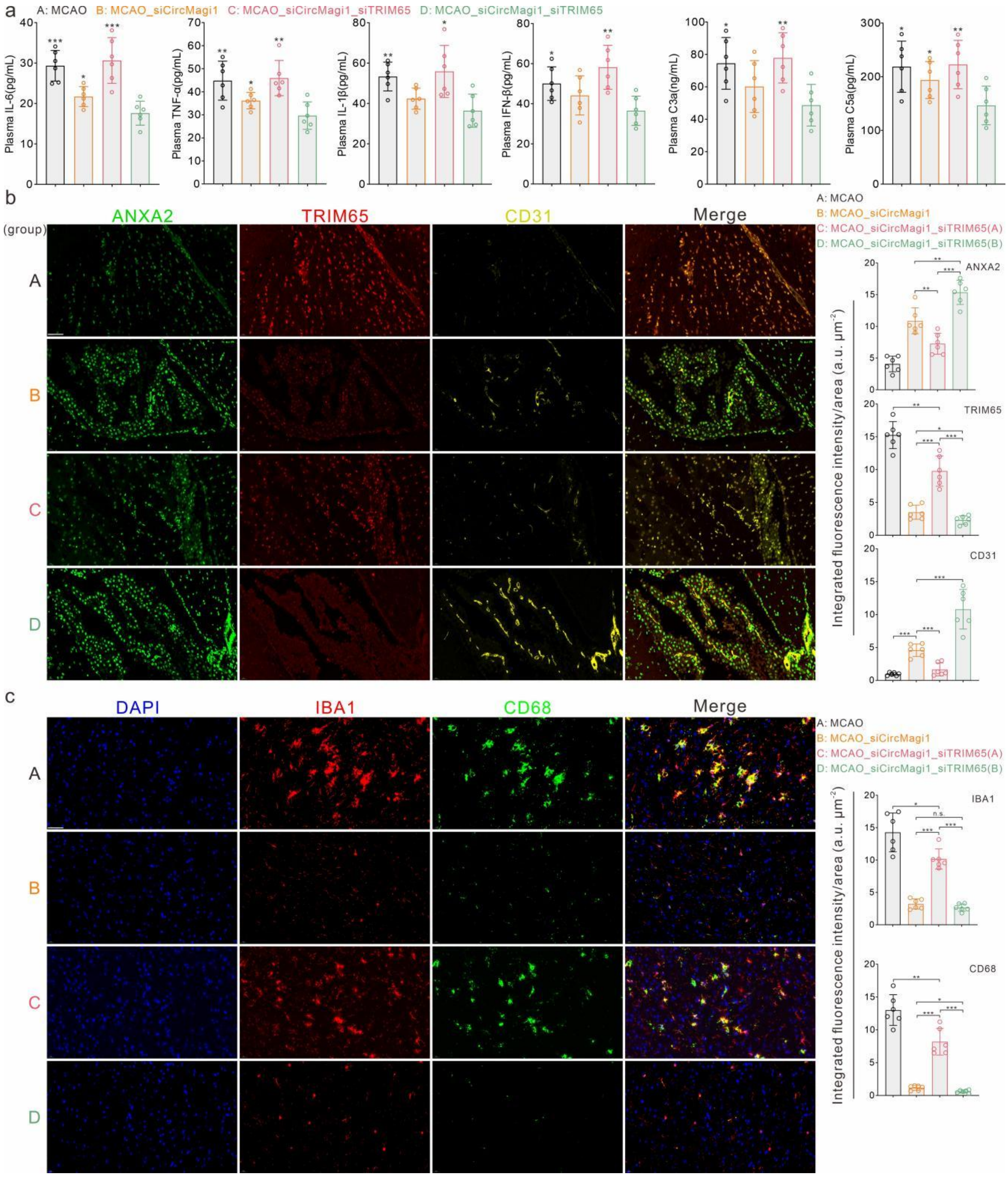

Fig. S7: Endpoint immune profiling and peri-infarct remodeling across TRIM65-inhibition schedules during sustained circMagi1 suppression. Related to Fig. 7.

(a) Plasma concentrations of IL-6, TNF-α, IL-1β, IFN-β, C3a, and C5a measured on post-MCAO Day 28. Group A comprised untreated MCAO mice. Group B received siCircMagi1-RVG-EVs every 4 days from Day 1 through Day 25. Groups C and D received the same siCircMagi1-RVG-EV regimen, with siTrim65-Ang2-EVs administered every 4 days from Day 1 or Day 9 through Day 25, respectively. (b) Representative peri-infarct immunofluorescence images of ANXA2, TRIM65, and CD31, together with the corresponding fluorescence-signal quantification. Scale bars, 20 μm. (c) Representative peri-infarct immunofluorescence images of IBA1 and CD68, together with the corresponding fluorescence-signal quantification. Each data point represents one mouse, with measurements from multiple fields averaged

to obtain one biological value per animal (n = 6 mice per group). Scale bars, 20 μm. Data are presented as mean ± s.d. Statistical significance was determined using one-way ANOVA followed by Tukey's multiple-comparison test. Significance is denoted by $^{*}P < 0.05$, $^{**}P < 0.01$, and $^{***}P < 0.001$.

Fig. S8a (Fig. 5c)

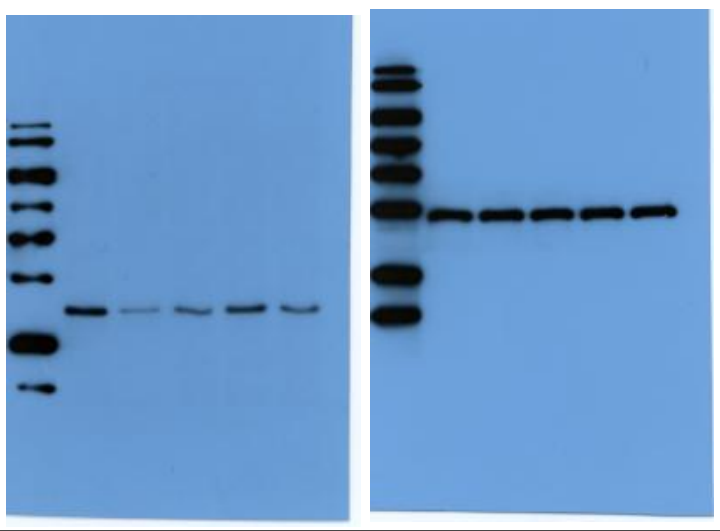

Fig. S8b (Fig. 5d)

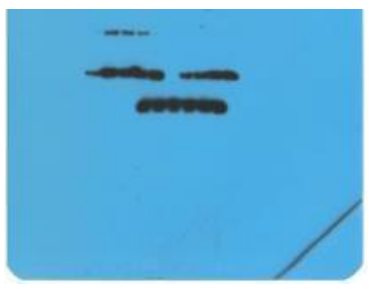

Fig. S8c (Fig. 5e)

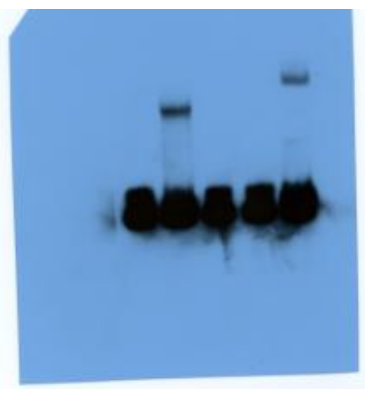

Fig. S8d (Fig. 5h)

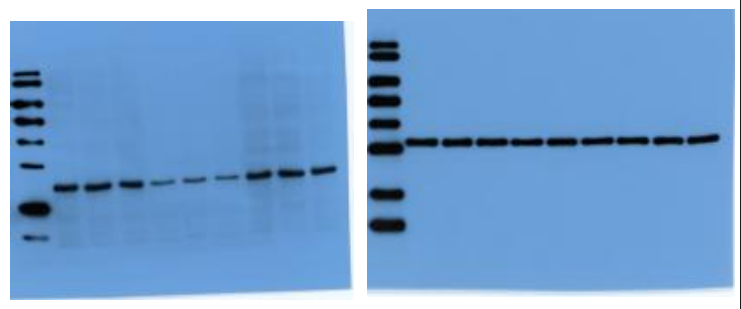

Fig. S8e (Fig. 6a)

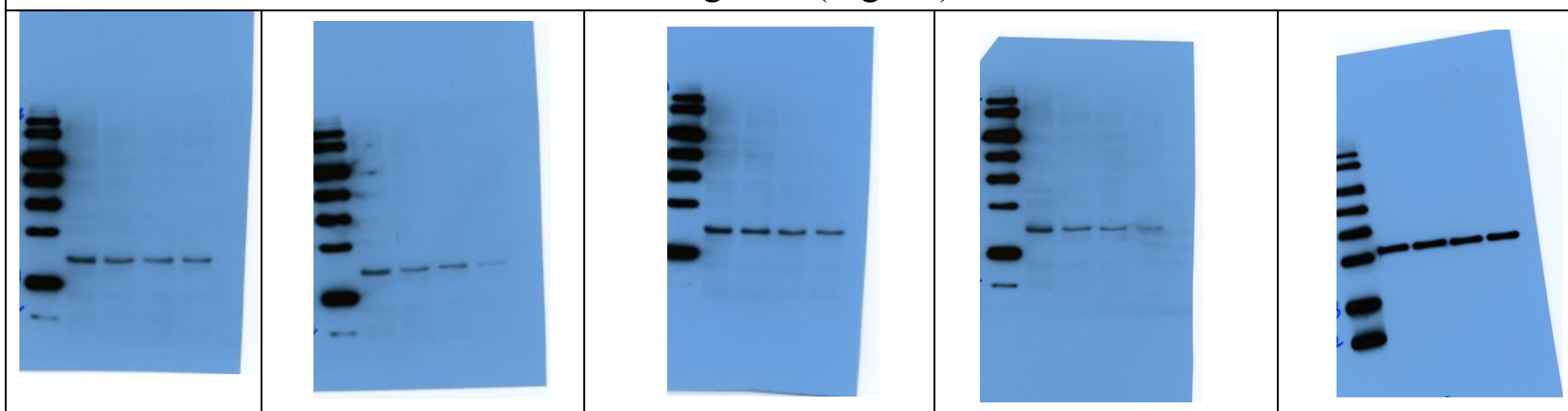

Fig. S8f (Fig. 6b)

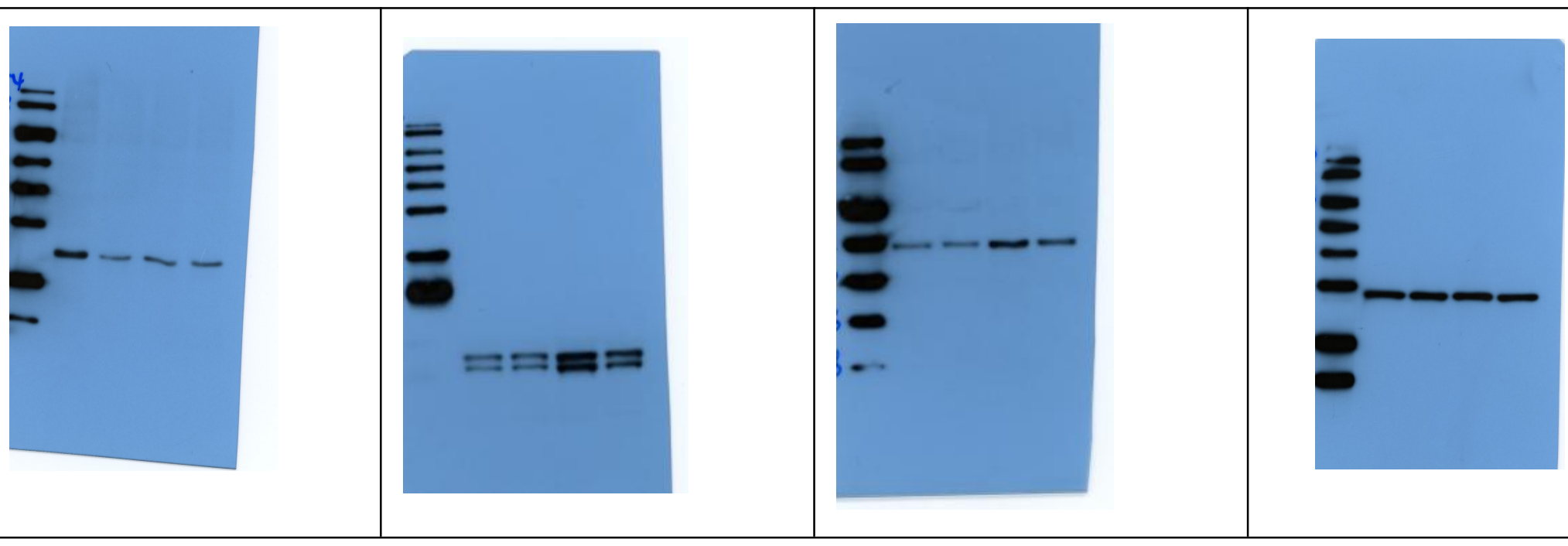

Fig. S8g (Fig. S6a)

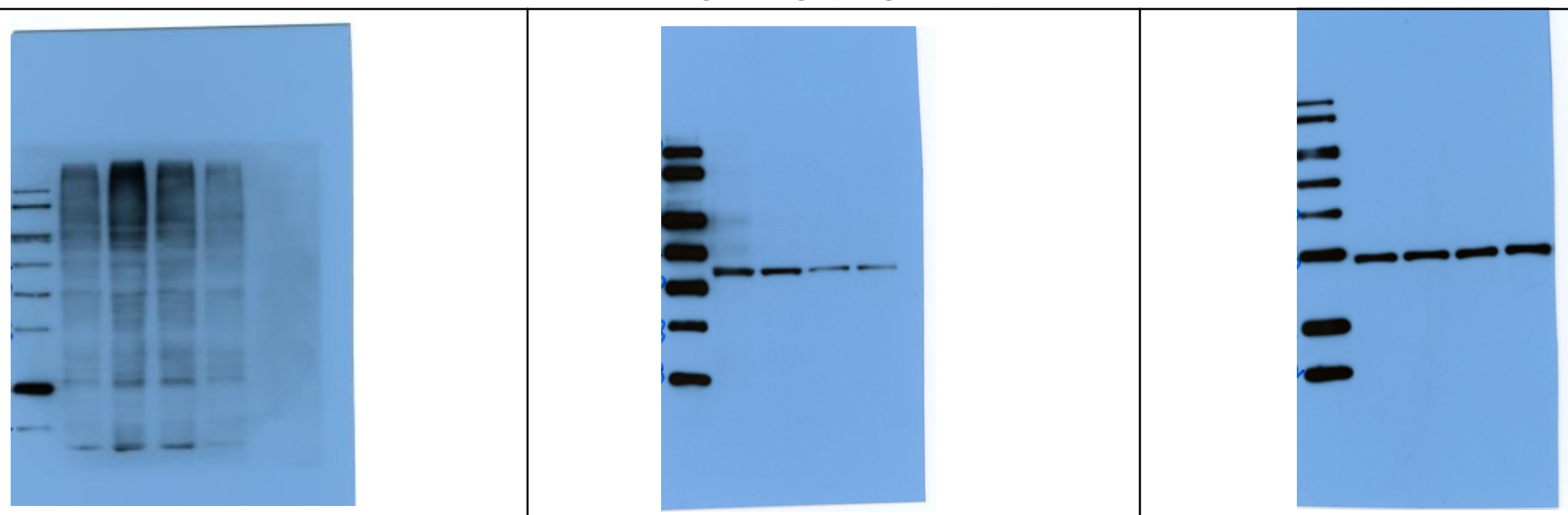

Fig. S8h (Fig. S6b)

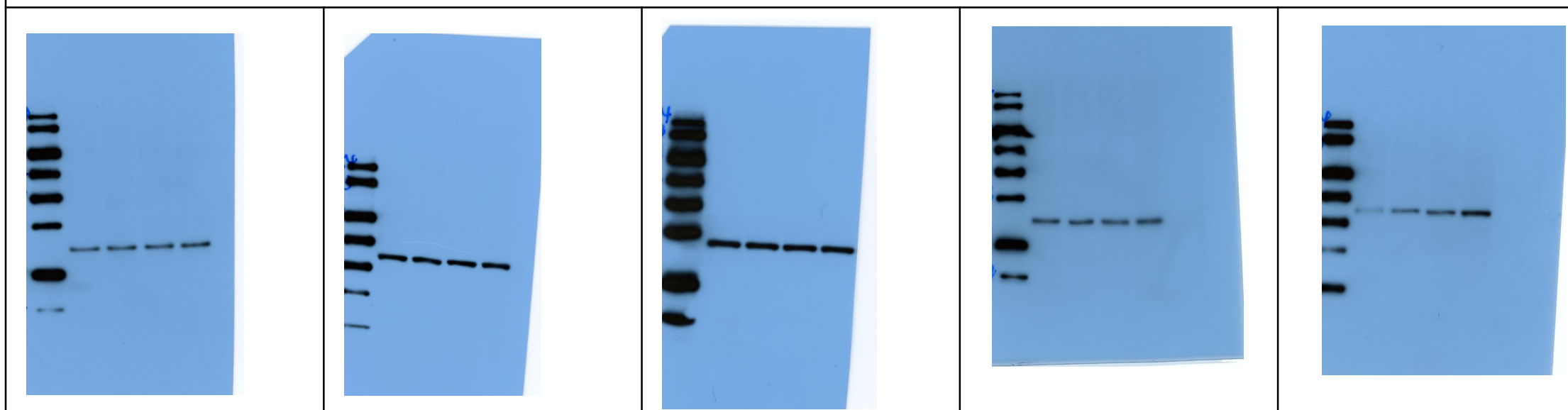

Fig. S8i (Fig. 6c)

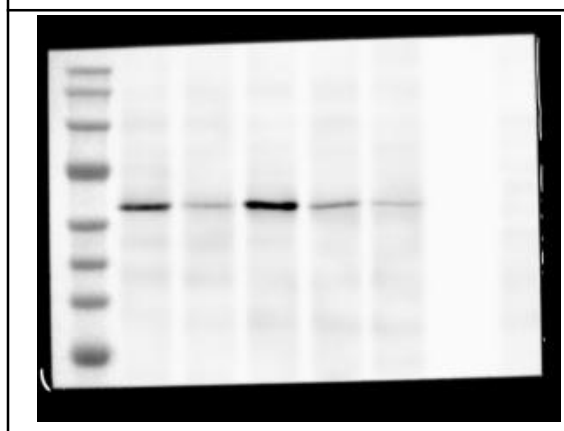
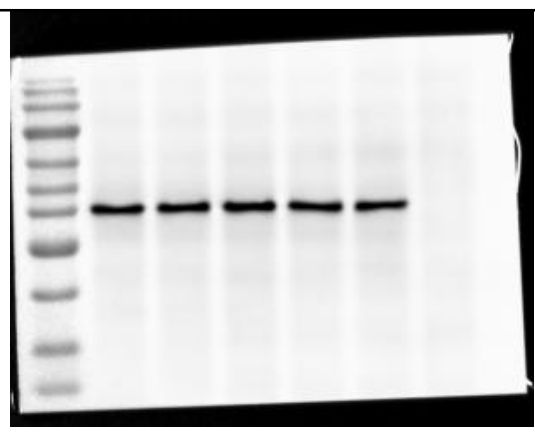
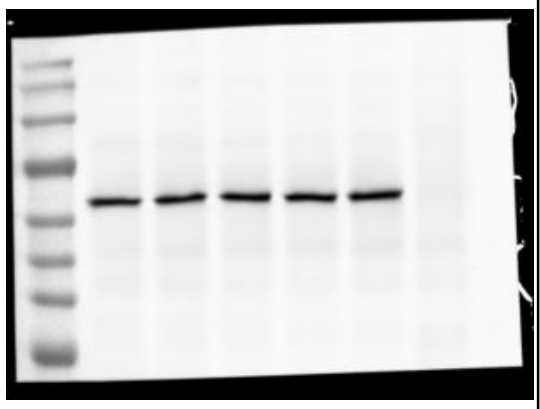
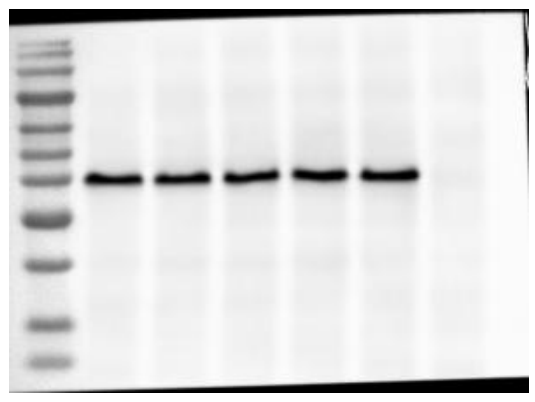

Fig. S8j (Fig. 6d)

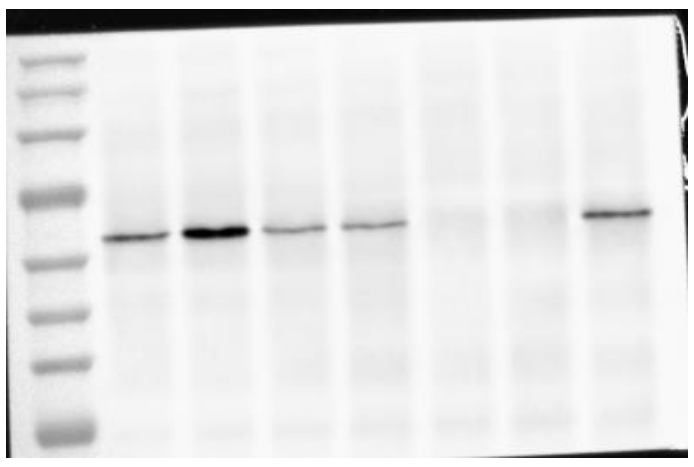
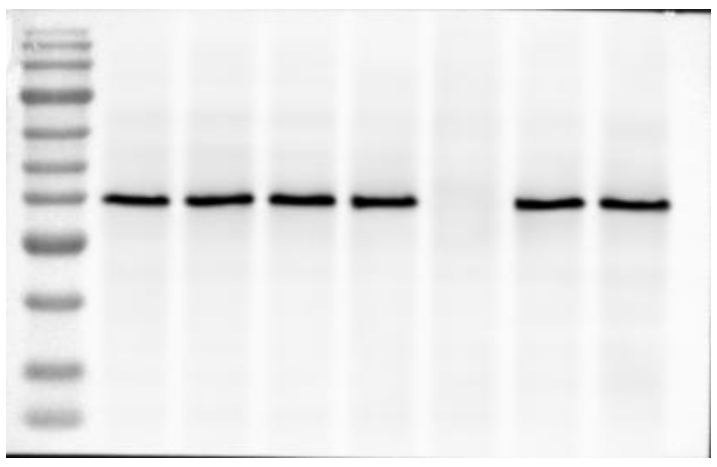
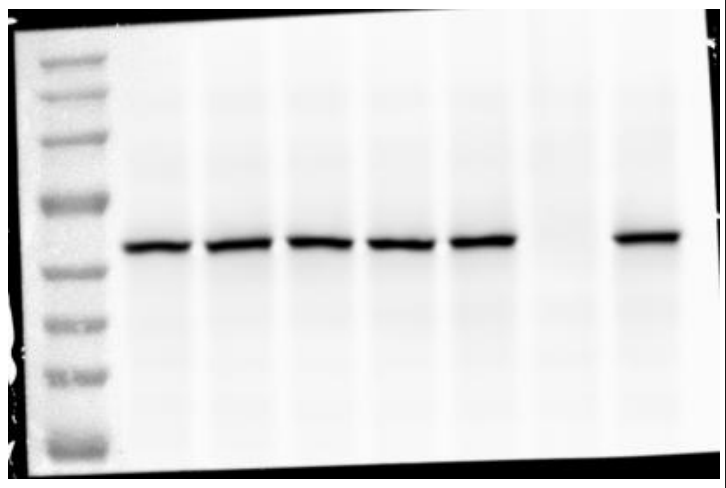

Fig. S8k (Fig. 6e)

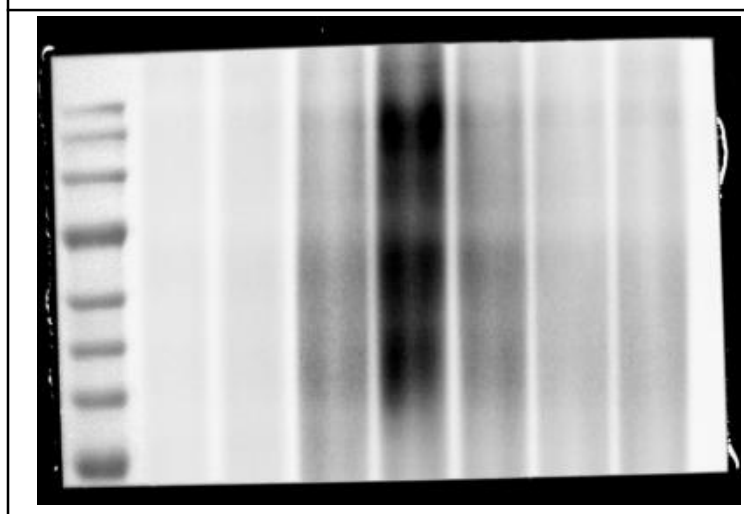
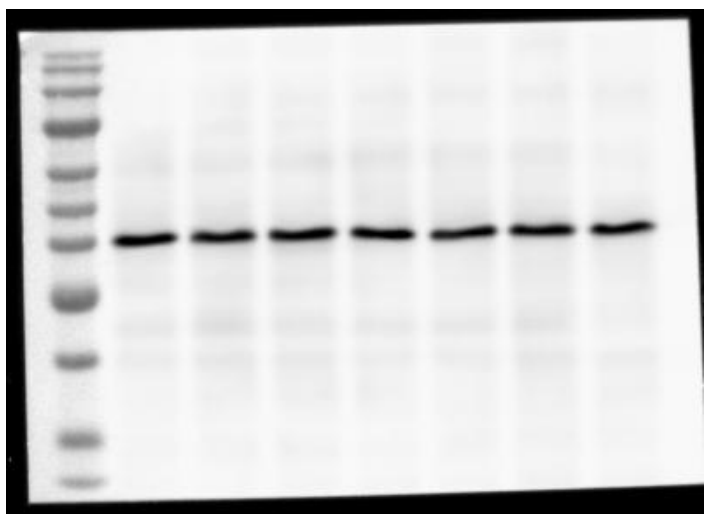
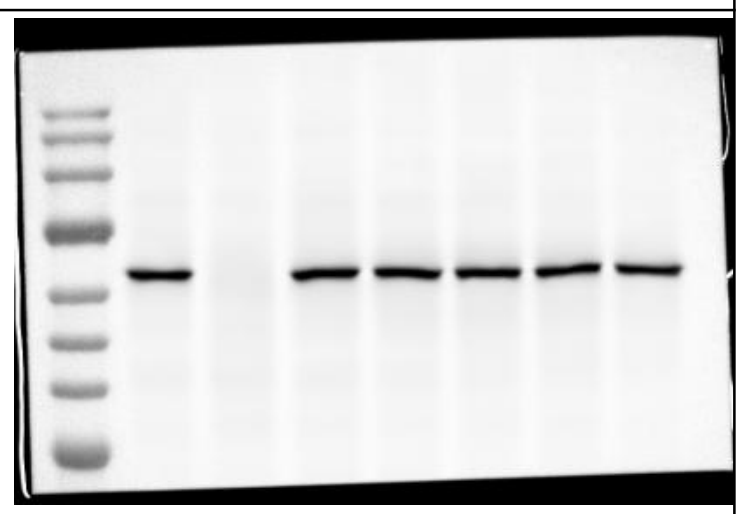

Fig. S8l (Fig. 6f)

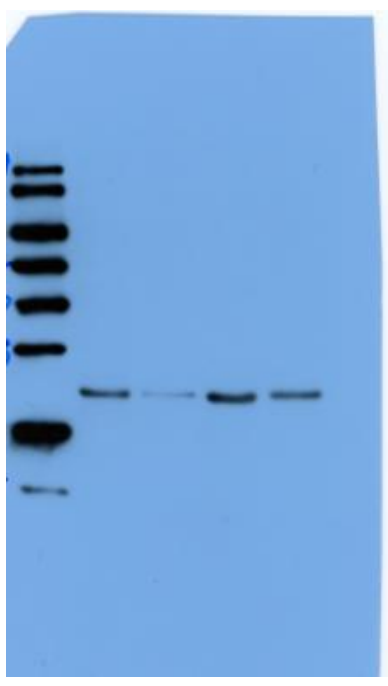
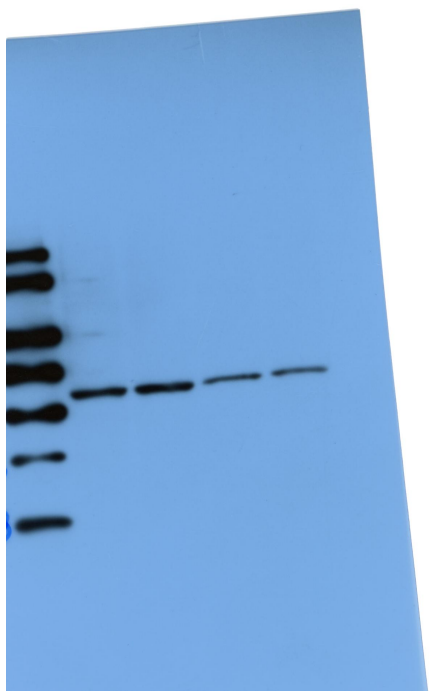
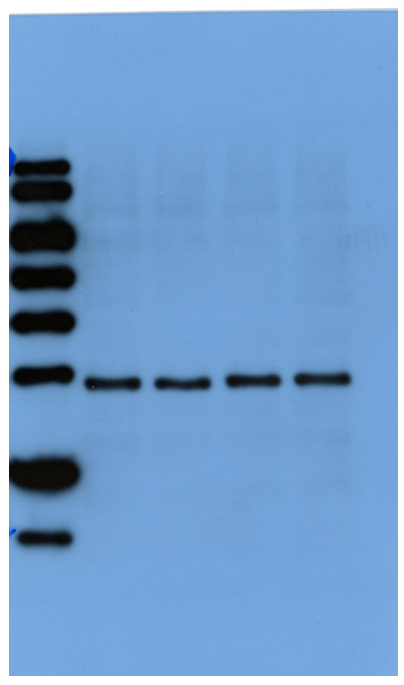

Fig. S8: Original, uncropped blot images. Related to Figs. 5, 6 and Fig. S6.
